# The neuroscience of highly stable, positive, and refined states of consciousness during jhana-type advanced concentration absorption meditation (ACAM-J)

**DOI:** 10.1101/2025.11.12.688050

**Published:** 2025-11-13

**Authors:** Winson F.Z Yang, Ruby Potash, Grace Mackin, Isidora Beslic, Marta Bianciardi, Terje Sparby, Matthew D. Sacchet

**Affiliations:** 1 Meditation Research Program, Department of Psychiatry, Massachusetts General Hospital, Harvard Medical School, Boston, USA; 2 Athinoula A. Martinos Center for Biomedical Imaging, Department of Radiology, Massachusetts General Hospital, Harvard Medical School, USA; 3 Northeastern University, Boston, USA; 4 Brainstem Imaging Laboratory, Department of Radiology, Athinoula A. Martinos Center for Biomedical Imaging, Massachusetts General Hospital and Harvard Medical School, Boston, MA, USA.; 5 Division of Sleep Medicine, Harvard University, Boston, MA, USA.; 6 Steiner University College, Oslo, Norway; 7 Department of Psychology and Psychotherapy, Witten/Herdecke University, Witten, Germany; 8 Integrated Curriculum for Anthroposophic Psychology, Witten/Herdecke University, Witten, Germany

**Keywords:** advanced meditation, advanced concentration absorption meditation-jhana type (ACAM-J), 7T functional magnetic resonance imaging (fMRI), neurophenomenology, human flourishing, well-being

## Abstract

Humans can experience a rich array of conscious experience, including highly stable, positive, and refined states of non-ordinary perception that can be elicited through advanced meditation. Here, we present the first group-level, ultra–high-field (7T) fMRI study of jhana advanced concentration absorption meditation (ACAM-J). We combined local (regional homogeneity), mesoscale (connectivity gradients), and global (geometric eigenmodes) human brain mapping with diverse and detailed phenomenology, meditative traits, behaviorally assessed cognitive functions, and extensive publicly available psychobehavioral affinity maps. Across eight successive states of ACAM-J, we observed reproducible neural trajectories marked by anterior-to-posterior reorganization, flattening of cortical hierarchies, nonlinear reconfiguration of global harmonics, and tight coupling between brain metrics and equanimity, attentional stability, and behavior. Neurosynth decoding further associate ACAM-J with reduced suffering-related processes and more with attentional monitoring. These findings suggest ACAM-J is a distinct, structured mode of awareness that persists despite radically reduced narrative thought and sensory content. Our findings also inform boundary conditions for current models of consciousness. More broadly, this research indicates that advanced meditation is a powerful framework for understanding psychological transformation, and for potential new opportunities for supporting human well-being and flourishing.

Human consciousness is not confined to ordinary perception and thought. With training, it can reorganize into highly phenomenologically structured, non-ordinary states of consciousness (1, 2). According to contemplative traditions, advanced meditation practices progress toward meditative endpoints (2) characterized by what are thought to be irreversible shifts in perception, identity, and cognition (3, 4), known variously as “enlightenment” (5, 6), “awakening” (7), or “salvation” (8). Rather than being driven by narrative processing and reactive affect and cognition, these states of consciousness cultivate a radically different organization of awareness rooted in equanimity, non-judgment, and openness (9–13). These states of consciousness are sometimes reported to be phenomenologically vivid despite the absence of ordinary narrative thought. These experiences remain almost entirely unstudied in modern science (14), despite their significance in contemplative traditions and their potential to inform both the upper limits of human potential and the mechanisms of psychological suffering (15, 16).

Unlike the heterogeneous effects of ordinary meditation, these advanced meditation states are structured and reproducible, with practitioners able to reliably enter and describe them (2). This makes advanced meditation states a unique natural model for testing deeper theoretical challenges of consciousness, including how the brain sustains phenomenally vivid awareness near absence of narrative thought, affect, and sensory content. Importantly, these states may strongly counteract the patterns of rumination, hyper-reactivity, compulsive craving that characterize psychiatric disorders, pointing to new frameworks for conceptualizing mental health, including well-being and flourishing.

Among the most structured and reproducible maps of advanced meditation described in contemplative traditions is *jhāna*, a sequence of absorption states preserved in Buddhism for over two millennia (17). Here, we refer to this practice scientifically as jhana advanced concentration absorption meditation (ACAM-J), thereby distinguishing the neurocognitive features under investigation scientifically from doctrinal interpretations. This thereby anchors in phenomenology and avoids, in some cases, thousands of years of controversies for example, related to defining jhana. ACAM-J are eight successive advanced meditation states that emerge through increasingly subtle absorption and attentional refinement (17). Early ACAM-J are characterized by strong positive affective qualities of joy, bliss, and energized yet stable attention. In contrast, later ACAM-J are characterized by a profound sense of formlessness, marked by the reduced sensory content and self-referential thought. Awareness becomes expansive, boundless, and subtle, often described as spacious emptiness or a state beyond ordinary perception. Each ACAM-J is phenomenologically distinct and reliably reportable by experienced practitioners (18). This makes ACAM-J a powerful and ideal framework for systematic investigation of non-ordinary consciousness. Despite this potential, ACAM-J remains unstudied in modern neuroscience, with prior studies constrained to limited methodology and single-case observations (14, 17, 19–27).

Contemporary models of consciousness such as the Global Neuronal Workspace Theory (GNWT) (28) and Integrated Information Theory (IIT) (29) emphasize sensory processing, task engagement, and narrative cognition, hypothesizing that conscious experience depends on information accessibility or integration to support goal-directed behavior. These models account well for ordinary experience but struggle to explain content-minimal yet phenomenologically vivid states like ACAM-J (30, 31). Recent proposals such as the Minimal Phenomenal Experience (MPE) (32) address this by characterizing sparse but present conscious states but remain untested against structured meditative trajectories. ACAM-J thus offers a unique test case for evaluating and extending theories of consciousness.

Here, we present the first group-level, multiscale neurophenomenological study of ACAM-J, combining ultra-high-field 7 Tesla fMRI we integrated local (regional homogeneity), mesoscale (connectivity gradients), and global (geometric eigenmodes) measures with detailed phenomenology, meditative traits, and behavioral assessments. Our findings reveal a reproducible neural trajectory of absorption marked by anterior-to-posterior reorganization, flattening of cortical hierarchies, nonlinear trends in global harmonics, and tight coupling to subjective equanimity and cognition. These results establish ACAM-J as a distinct, structured mode of awareness with a unique neural signature that challenges current models of consciousness and opens new avenues for the science of consciousness, wellbeing, and psychiatry.

## Results

Given that the two control conditions (counting and memory) showed minimal differences for regional homogeneity (ReHo), connectivity gradients, and geometric eigenmodes, we averaged them for primary comparisons with ACAM-J. Full control-versus-control analyses and extended ROI tables are provided in the supplementary materials.

### Brain activity: ReHo

**Fig. 1.**
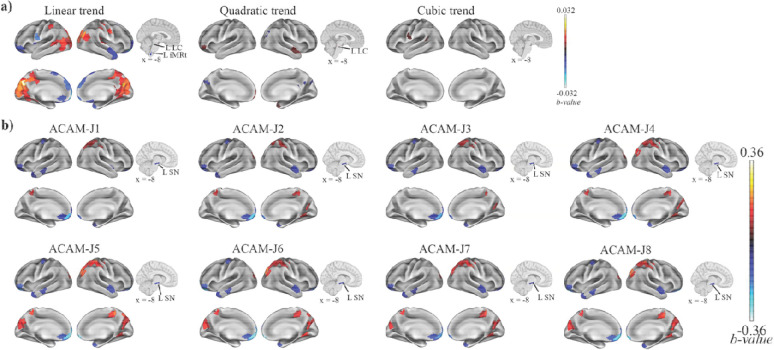
Distinct regional homogeneity (ReHo) during advanced concentration absorption meditation called jhana (ACAM-J). (a) Trends analyses show regions exhibiting linear, quadratic, and cubic trends across task ACAM-J. Linear trends highlights progressive and linear increase or decrease in specific brain regions while quadratic trends show mostly negative (inverted U-shaped) changes across ACAM-J. Positive but small cubic trend was found in the left somatomotor and parietal cortex. (b) ReHo differences between ACAM-J and the composite control reveal distinct patterns of cortical and brainstem substantia nigra activity.

Comprehensive results/summary tables of significant regions are included in the **supplementary excel**. Here, we only provide a summary of the results. Across the eight ACAM-J, ReHo revealed systematic reorganization of brain activity (Fig. 1a), with increasing trends in posterior regions (bilateral visual cortex, precuneus–posterior cingulate, parietal, and somatomotor cortices), and decreasing trends in anterior and subcortical hubs (bilateral prefrontal [PFC] and orbitofrontal [OFC] cortices, brainstem regions associated with autonomic function (locs coeruleus, inferior medullary reticular formation). Higher-order polynomial fits revealed additional non-linear modulations, with quadratic trends in midline regions, and isolated cubic effects in posterior regions. Compared with the control conditions, ACAM-J showed reductions in prefrontal, orbitofrontal, temporal cortices and substantia nigra (SN, associated with arousal), and increase ReHo in posterior sensory regions (Fig. 1b). These changes followed a state-dependent trajectory: early ACAM-J were dominated by anterior and subcortical decreases, whereas later ACAM-J (J7–J8) displayed pronounced increases in visual and somatomotor cortices.

### Macroscale hierarchy: Principal connectivity gradients

**Fig. 2.**
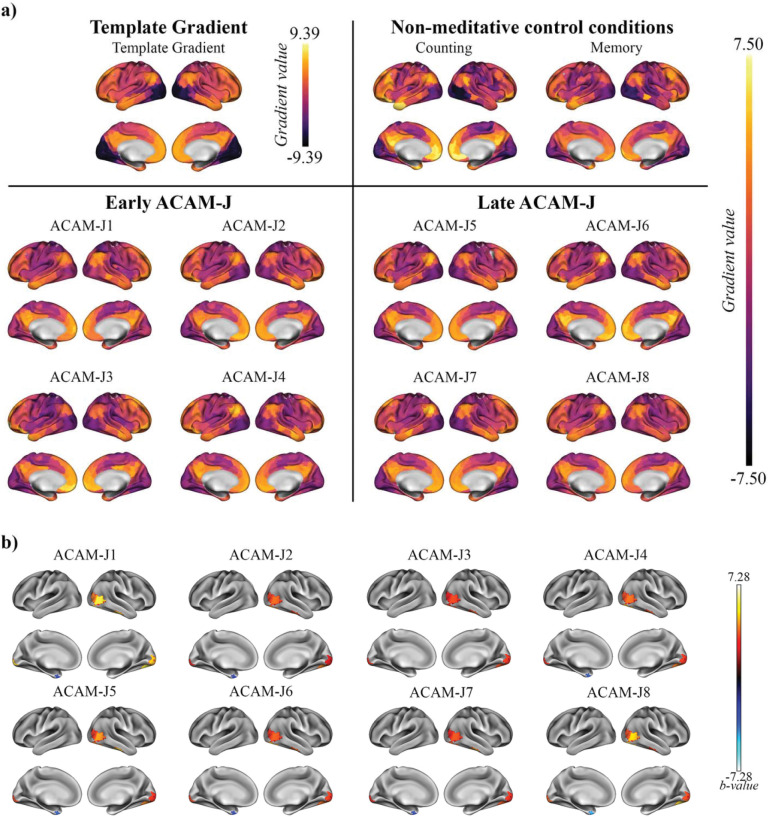
Principal connectivity gradient (G1) demonstrates functional hierarchical neural organization during ACAM-J. (a) Template principal gradient (G1) used for gradient alignment in this study, and average gradient maps for the non-meditative control conditions (counting, memory) and ACAM-J, grouped into early and late absorption phases. (b) Contrasts between individual ACAM-J and the composite of control conditions reveal robust changes along G1 in visual and temporal hubs highlighting regional-specific reorganization of large-scale cortical hierarchy during ACAM-J.

The principal connectivity gradient (G1) maintained a canonical unimodal-to-transmodal hierarchy during control tasks but was markedly flattened during ACAM-J (Fig. 2a). No significant trends were observed throughout ACAM-J. However, when compared directly to control conditions, ACAM-J was consistently associated with higher G1 values in visual and right temporal cortices and lower values in the left temporal pole (Fig. 2b). This pattern indicates that while hierarchical organization is globally compressed, regional reconfigurations are non-uniform and potentially reflect altered functional specialization during absorption. Full ROI-level results are reported in Supplementary Table S3.

### Global cortical dynamics: Geometric eigenmodes

**Fig. 3.**
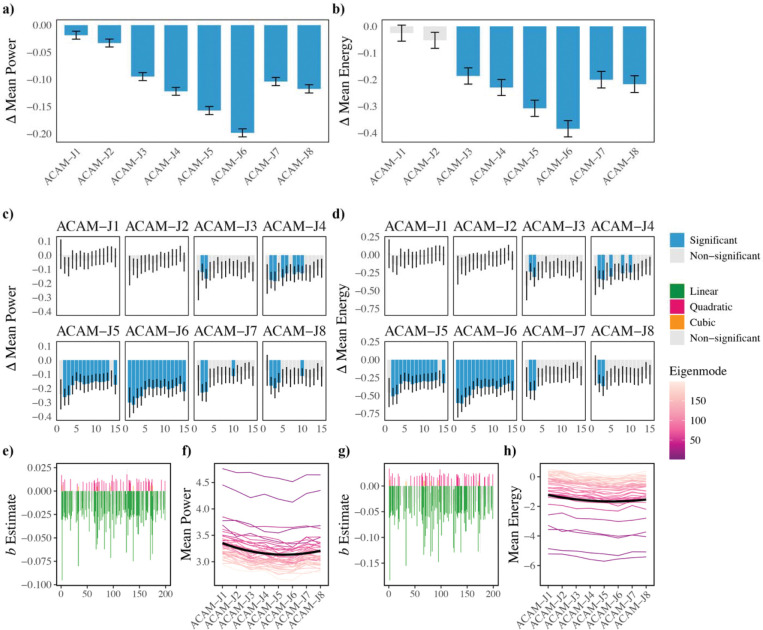
Geometric eigenmode decomposition reveals large-scale cortical dynamics during ACAM-J relative to non-meditative control conditions. Mean eigenmode power (a) and energy (b) during ACAM-J relative to the composite control condition. Eigenmode spectra for individual eigengroups highlight systematic reductions in power (c) and energy (d) during ACAM-J compared to control conditions, with strongest effects during mid-to-late ACAM-J. Plots (e, g) show distribution of significant trends across the eigenmodes indicate predominantly negative linear effects, reflecting progressive decreases with increasing absorption depth, along with a smaller number of quadratic effects. Plots (f, h) demonstrate changes in mean power (f) and mean energy (h) across ACAM-J, revealing positive quadratic trends: decreases in power and energy up to mid-ACAM-J, followed by recovery and increases through ACAM-J8. Error bars represent standard errors.

Overall mean power/energy were significantly lower during ACAM-J compared with controls, except for mean energy of ACAM-J1 and ACAM-J2 (Fig. 3a–b). Frequency-specific analyses showed the most remarkable differences between ACAM-J5–6 and control conditions (Fig. 3c–d). No significant interactions between eigenmodes and ACAM-J were observed (p > 0.05). In contrast, polynomial trend analyses uncovered a robust trajectory across ACAM-J. Both mean power/energy exhibited consistent negative linear and positive quadratic trends, indicating an early decline from ACAM-J1 through ACAM-J5 followed by recovery in later ACAM-J (Fig. 3e–h). Although both metrics followed the same U-shaped pattern, the quadratic effects were less pronounced for energy than for power. Together, these results point to a nonlinear reconfiguration of large-scale cortical harmonics during absorption, characterized by an initial compression of global dynamics followed by re-expansion in advanced absorption. Full polynomial coefficients and eigenmode-level statistics are provided in Supplementary Tables S3.

### Multivariate and univariate brain-experience relationships: Linking neuroimaging metrics with phenomenology, meditative traits, and cognition

To examine how neural dynamics relate to subjective and behavioral measures, we applied partial least squares correlation (PLSC) analyses linking ReHo, gradients, and eigenmodes with phenomenology, meditative traits, and cognition. For clarity, we report only the leading latent variables in the main text; full details of secondary LVs are provided in the Supplementary results.

#### Brain activity: ReHo.

**Fig. 4.**
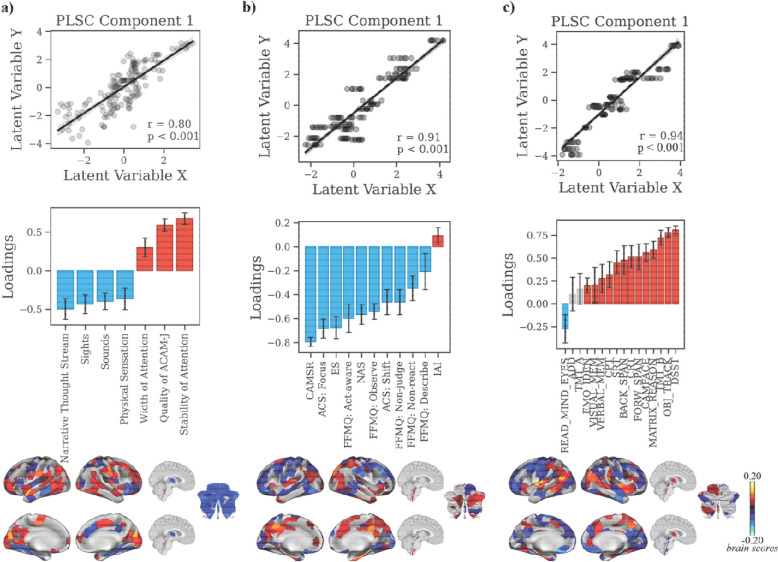
Multivariate relationship between ReHo during ACAM-J and behavioral patterns (ACAM-J phenomenology, meditative characteristics, and general behavioral performance). Partial least squares correlation (PLSC) analyses linking regional homogeneity (ReHo) during ACAM-J to three behavioral domains: (a) phenomenological ratings, (b) meditative characteristics, and (c) general behavioral performance. For each analysis, scatterplots of the first latent variable (top) show significant brain–behavior correlations (all p < 0.001). Bar plots (middle) display behavioral loadings, with error bars denoting bootstrap-estimated confidence intervals. Brain maps (bottom) depict regional ReHo loadings contributing to each multivariate pattern. Error bars represent standard errors.

For ACAM-J phenomenology (39.52% shared covariance, p_FDR_ < 0.001; r = 0.80, p < 0.001; Fig. 4a), posterior sensory–attentional regions (visual, somatomotor, precuneus/posterior cingulate) loaded positively with correspondence of ACAM-J, stability, and width of attention, while anterior control and medial-temporal higher-order cognitive regions (medial/anterior prefrontal, ventral temporal/hippocampal, cerebellar), basal ganglia, and cerebellum loaded negatively with sensory experiences and narrative thought (Fig. 4a). Meditative traits (42.32% shared covariance, p_FDR_ < 0.001; r = 0.91, p < 0.001; Fig. 4b) showed primarily negative loadings, except for interoceptive awareness. This pattern was associated with positive weights in the medial frontal and posterior regions, temporal poles, and subcortical hubs (brainstem), and negatively in the lateral PFC, and visual regions. With cognition (27.27% shared covariance, p_FDR_ < 0.001; r = 0.94, p < 0.001; Fig. 4c), general cognitive performance (working memory, sustained attention, executive control) loaded positively with temporoparietal, dorsomedial PFC regions, and basal ganglia, while social cognition loaded negatively with lateral PFC, OFC, visual cortex, and brainstem.

#### Macroscale hierarchy: Principal gradients.

**Fig. 5.**
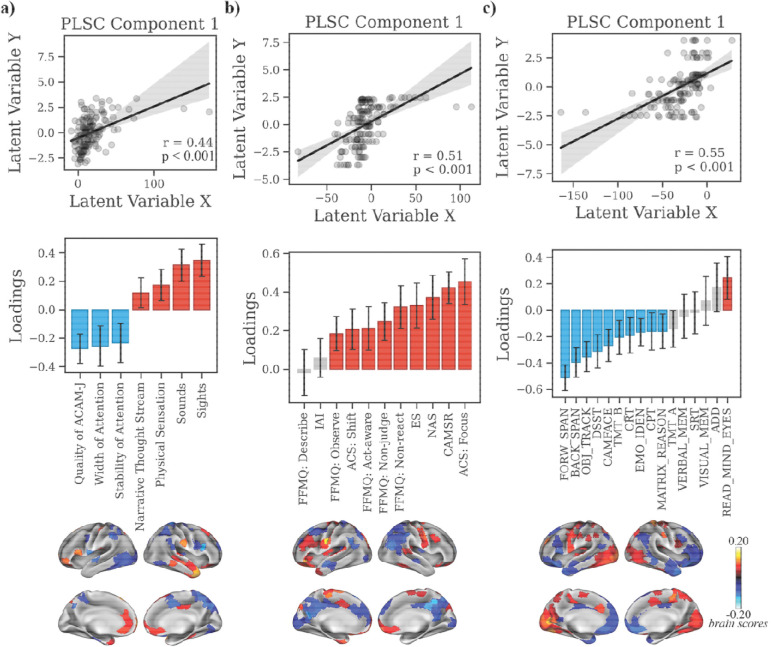
Multivariate relationship between G1 during ACAM-J and behavioral patterns (meditative characteristics, and general cognitive performance). Partial least squares correlation (PLSC) analyses linking principal gradient values (G1) during ACAM-J to three behavioral domains: (a) phenomenology, (b) meditative characteristics, and (c) general cognitive performance. For each analysis, scatterplots of the first latent variable (top) show significant brain–behavior correlations (all p < 0.001). Bar plots (middle) display behavioral loadings, with error bars denoting bootstrap-estimated confidence intervals. Brain maps (bottom) depict regional G1 loadings contributing to each multivariate pattern.

Phenomenologically (49.27% shared variance, p_FDR_ = 0.018; r = 0.44, p < 0.001; Fig. 5a), higher G1 values in transmodal regions (medial/lateral PFC and anterior temporal regions) was associated with higher sensory vividness, whereas reduced unimodal loadings (visual, somatomotor, posterior parietal) were associated with attentional absorption (width of attention, stability of attention, and correspondence of ACAM-J). Meditative trait associations (40.75% shared variance, p_FDR_ = 0.0016; r = 0.51, p < 0.001; Fig. 5b) loaded positively on almost all traits, which covaried with loadings in transmodal regions (medial/lateral PFC, temporal poles), from non-significant descriptive/interoceptive awareness, which loaded on unimodal sensorimotor/parieto-occipital regions. For cognitive performance (38.29% shared variance, p_FDR_ < 0.001; r = 0.55, p < 0.001; Fig. 5c), social cognition mapped to sensorimotor/temporoparietal/visual regions, while general performance loaded on medial PFC and anterior temporal regions.

#### Global cortical dynamics: Geometric eigenmodes.

**Fig. 6.**
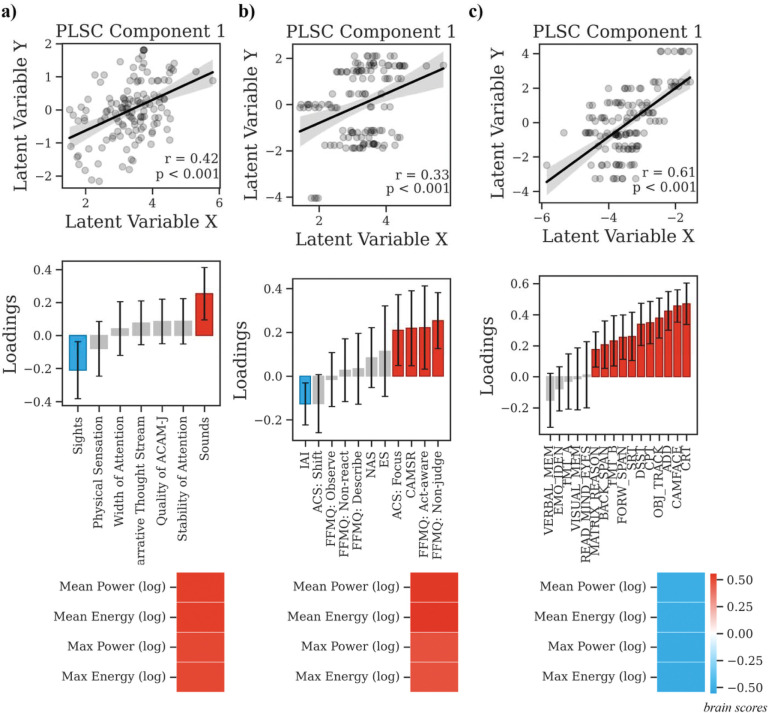
Multivariate relationship between global eigenmode activations during ACAM-J and behavioral patterns (meditative characteristics, and general cognitive performance). Partial least squares correlation (PLSC) analyses linking global eigenmode activations during ACAM-J to three behavioral domains: (a) phenomenology, (b) meditative characteristics, and (c) general cognitive performance. For each analysis, scatterplots (top) of the respective latent variable show significant brain–behavior correlations. Bar plots (middle) display behavioral loadings, with error bars denoting bootstrap-estimated confidence intervals. Heatmaps (bottom) show the global eigenmode activation loadings contributing to each multivariate pattern.

Global spectral activation covaried positively together with sensation of sounds and oppositely phenomenology of sights and negative (97.00% shared variance, p_FDR_ < 0.001; r = 0.42, p < 0.001; Fig. 6a). In the meditative traits domain, global spectral activation positively linked to non-judgment and focus (98.47% shared variance, p_FDR_ = 0.006; r = 0.33, p < 0.001; Fig. 6b). In the cognitive domain, higher cognitive performance was associated with lower global spectral activation (99.46% shared variance, p_FDR_ < 0.001; r = 0.61, p < 0.001; Fig. 6c).

#### Univariate brain-experience for ACAM-J factors.

For ReHo, equanimity was selectively associated with reduced ReHo in the right hippocampus, while formlessness showed positive associations with temporal regions, and negative associations with prefrontal, precuneus-posterior cingulate cortex, PFC, and somatomotor cortex. For principal gradients, formlessness was positively associated with G1 values in the right medial cortex, and mean power/energy showed no associations with ACAM-J factors.

#### Psychobehavioral affinity map decoding.

**Fig. 7.**
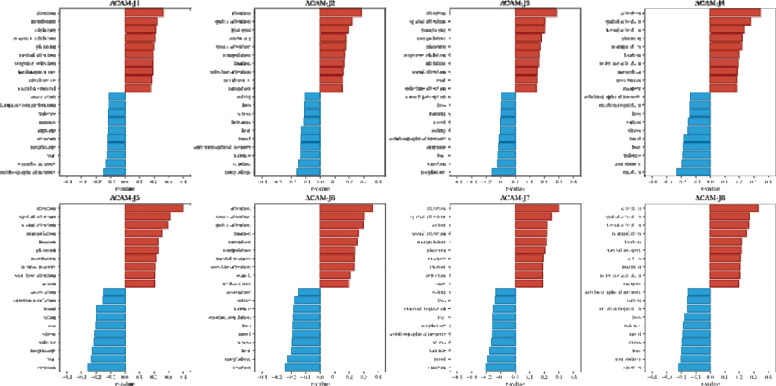
Reorganization of psychobehavioral domains during ACAM-J. For each ACAM-J, bar plots display the top ten functions with positive (red) and negative (blue) associations derived from Neurosynth decoding. Positive associations were dominated by attentional, executive, visuospatial, and action-related domains (e.g., sustained and selective attention, planning, imagery). In contrast, negative associations consistently involved affective, autobiographical, and bodily–social domains (e.g., emotion, mood, stress, fear, autobiographical memory, social cognition, eating, morphology). This dissociation highlights a robust psychobehavioral reorganization during ACAM-J, with enhanced attentional–executive functions accompanied by down-regulation of affective and self-referential processes.

Across ACAM-J, we observed a highly consistent functional dissociation between attentional–executive versus affective–autobiographical psychobehavioral affinity maps (Fig. 7). Functions with positive associations with ACAM-J were dominated by attentional and control-related processes, including sustained and selective attention, spatial and visual attention, planning, action, and mental imagery. These domains were among the strongest and most consistent effects across all ACAM-J, indicating systematic engagement of executive and visuospatial control networks. In contrast, functions with negative associations with ACAM-J clustered around affective, autobiographical, and bodily–social processes, including emotion, mood, valence, fear, stress, loss, and anxiety, as well as autobiographical and episodic memory, social cognition, and eating. This pattern suggests systematic down-regulation of affective and self-referential functions during ACAM-J. Moreover, the magnitude of the correlations were larger as ACAM-J deepens toward ACAM-J8.

## Discussion

This study provides the first multidimensional neuroscientific characterization of ACAM-J in a deeply phenotyped fMRI cohort (N= 20). Using ultra-high-field 7T fMRI and multiscale analyses, we show that ACAM-J constitutes a distinct, structured mode of consciousness marked by reproducible trends in local brain activity, macroscale gradients, global eigenmodes, and their associations with phenomenology, meditative traits, behavioral assessments, and psychological brain maps. Our findings reveal not only the capacity of the brain to volitionally sustain altered states of consciousness, but also its potential to evolve into structured, non-ordinary modes that align with meditative endpoints. These transformations may represent adaptive neurocognitive mechanism that support mental balance, clarity, and well-being, pointing to the brain’s endogenous capacity for human flourishing.

Using ReHo as an index of brain activity, we revealed a systematic redistribution of local activity: prefrontal and orbitofrontal hubs associated with narrative thought and cognitive control were progressively down-regulated, while posterior sensory and midline hubs (visual, somatomotor, precuneus/posterior cingulate) became more active. Furthermore, brainstem regions associated with arousal (locus coeruleus) and autonomic function (inferior medullary reticular formation) showed reduced activity throughout ACAM-J. We replicated findings from our previous intensive case study, demonstrating that early ACAM-J is characterized by lower activity frontal regions, while later ACAM-J is characterized by enhanced posterior sensory activity (14). Here we show that anterior-to-posterior reorganization reflects a transition from conceptual, effortful processing (33–36) toward refined, subtle, and open awareness (14, 37). Moreover, the progressive reduction of activity in the locus coeruleus and inferior medullary reticular formation suggests a gradual disengagement of sympathetic autonomic activity (38), consistent with the transition toward equanimity and diminished bodily awareness characteristic of later ACAM-J (18). Together, ACAM-J may be understood as producing a state-specific reallocation of resources away from conceptual elaboration toward stabilized sensory-attentional processing.

To probe the low-dimensional architecture of functional connectivity during ACAM-J, we assessed principal connectivity gradients. Connectivity-gradient analyses indicated that while the canonical unimodal–transmodal axis was preserved during control tasks, it was markedly flattened during ACAM-J. Across ACAM-J, principal gradient undergoes a systematic compression, indicating a progressive collapse of hierarchical differentiation between unimodal and transmodal systems (25). Importantly, the effect was not globally uniform: specific regions such as visual and temporal cortices shifted upward along the gradient, while the left temporal pole shifted downward. This non-uniform reconfiguration may reflect the brain’s transition from a modular architecture toward a globally diffused yet functionally specialized coherent, integrative conscious field that may support stable, non-conceptual awareness (39, 40).

Complementing this at the global scale, geometric eigenmode analysis revealed a U-shaped trajectory across ACAM-J where early ACAM-J were characterized by compression of spectral power and energy, followed by re-expansion in later ACAM-J. This dynamic reconfiguration suggests that absorption transiently downregulate cortical harmonics before reintegration at a higher level of coordination. This brain dynamic may reflect a brain mode that support focused attention, mental stillness, and reduced sensory processing typical of absorption in meditation (41, 42). Surprisingly, our findings contrast with our previous case study (27) and also a prior studies involving psychedelic substances (43, 44), which both reported expansion and increased activation cortical brain mode.

One possible explanation for this discrepancy could be that the current findings are based on group-level data, capturing population-wide patterns that may smooth over individual variability, whereas the previous case study focused on a single individual, potentially reflecting idiosyncratic dynamics. One could also argue that studies involving psychedelic substances have a similar number of participants to our study with ACAM-J. However, a key difference between these studies and ours lies in the range of eigenmodes used for analysis. While research on connectome eigenmodes typically explores a range of up to 10,000 eigenmodes (43, 44), our study focuses on geometric eigenmodes, using a range of up to 200, in line with standard methodology (45). As a result, the lower activation energies captured through geometric eigenmodes in our study may not represent the higher activation energies observed in the higher eigenmodes associated with psychedelic studies. Nevertheless, our findings may reflect a controlled reintegration of complexity, suggesting that similar altered states of consciousness can emerge via distinct neurodynamic pathways.

Strikingly, multivariate analyses using PLSC demonstrate that ACAM-J is not reducible to isolated neural or psychological effects but reflects an integrated reorganization of brain–mind dynamics. Posterior, parietal, and sensory hubs appear to support attentional qualities, while prefrontal systems contribute to the suspension of narrative and sensory processing. This was evident in both mesoscale activity and macroscale hierarchy of the brain, and eigenmode dynamics suggests that the brain is reconfigured to sustain enduring meditative traits. These convergences establishes the neurophenomenology of ACAM-J (46) where phenomenology, traits, behavior, and neural signatures converge to a unified phenomenon. More importantly, the neural configurations suggest a disengagement from self-referential processing and ordinary perception, marking a transformative shift toward enhanced sensory and attentional processing (47, 48). This reorganization reflects a more flexible, refined engagement with the present moment, and moves beyond ordinary narrative-driven cognition to a state of heightened, dynamic sensory awareness.

Beyond neurophenomenology, we also examined the psychobehavioral affinity domains most associated with ACAM-J using Neurosynth decoding. A striking and robust dissociation emerged in which attentional and executive domains were consistently positively associated with brain activity during ACAM-J, while negative affect, autobiographical, and self-referential domains were systematically downregulated. This functional reorganization indicates that ACAM-J is associated with a structured shift toward enhanced attentional stability and reduced negative affect and self-referential processing. Importantly, this mirrors practitioners’ first-person reports of effortless attentional absorption and awareness, bliss, happiness, and equanimity. These results emphasize ACAM-J not as a passive withdrawal of consciousness but an active reconfiguration of neural dynamics toward sustained attention and perceptual clarity, accompanied by systematic down-regulation of affective–autobiographical functions. Thus, our neurophenomenological findings highlights the value of neurophenomenology in studying non-ordinary states of consciousness such as ACAM-J with the same rigor as perception or memory (49–52).

Our findings directly contest leading models of consciousness. GNWT proposes that consciousness depends on ignition across a prefrontal–parietal “rich club” (28). This ignition process enables flexible access, working memory, and goal-directed cognition (53). In this view, conscious states should be accompanied by widespread prefrontal–parietal activation and recurrent signaling through thalamocortical loops (54, 55). Yet, our findings directly challenge this assumption. ACAM-J, and more specifically higher ACAM-J, marked by minimal content, was associated with prefrontal downregulation and increased posterior–midline activity. Rather than broadcasting through a prefrontal workspace, consciousness here persisted in the posterior regions. Importantly, ACAM-J is not characterized by dullness or reduced awareness. ACAM-J involve heightened awareness, clarity, and even refined forms of metacognition, indicating a state of open yet focused consciousness (56).

This combination of expansive awareness and highly refined attentional stability appears paradoxical within standard cognitive frameworks, which often treat focused attention and open awareness as mutually exclusive. Such paradoxes may instead reveal the limits of current theories in explaining absorptive states, rather than contradictions in the phenomenology itself. This suggests that while GNWT may capture consciousness in the service of ordinary cognition, it does not account for states of conscious awareness without prefrontal broadcasting. In this sense, ACAM-J exposes a boundary condition for GNWT, that prefrontal “workspace” activity may not be necessary for consciousness per se, but rather perhaps for consciousness in service of cognitive control.

Similarly, IIT proposes that the level and richness of consciousness depends on the amount of integrated information a system can generate (29). A system is most conscious when it is both highly differentiated and highly integrated (57, 58). Our findings challenge the basic principle of this model. During ACAM-J, cortical gradients become diffused, indicating reduced differentiation between unimodal and transmodal systems. At the same time, geometric eigenmodes showed coherent dynamics in a low-dimensional space. Phenomenology remained vivid despite this reduction in modular complexity. One interpretation is that while the quantity of content to be integrated decreases, the quality of integration among the remaining processes is exceptionally strong. This results in an integration high in absorption and unity greater than in ordinary experiences (56). Our results suggest that consciousness does not always scale linearly with differentiation and integration. Instead, consciousness of absorption states such as those accompanying bliss, openness, and awareness of subtle qualities of the mind may emerge from simplified yet strongly coordinated neural signatures. From this perspective, ACAM-J both challenges and partly aligns with IIT, indicating that integration can sustain vivid consciousness even when differentiation is low.

MPE describe states in which consciousness is preserved in its most reduced form and are devoid of narrative thought, sensory imagery, and affective tone, yet still minimally aware (32). ACAM-J supports this view but extends it by showing a structured, trainable trajectory of content-minimal states unfolding in reproducible neural reorganizations. Unlike passive or spontaneous MPE, ACAM-J offers a progressive refinement of consciousness into such states. Our data supports this by showing dominant posterior activity, reduced prefrontal activity, and their associations with ACAM-J phenomenology. Together, these results suggest that consciousness is not bound to ignition or complexity, but can reorganize into globally coherent and open modes, offering GNWT and IIT boundary conditions and an extension for MPE.

The implications extend beyond the contemplative sciences. The neural correlates of ACAM-J may have profound consequences for our understanding of mental health, well-being, and the potential for human transformation (15, 16). The ability to selectively intensify executive functions and self-generate bliss and joy while downregulating emotional reactivity, autobiographical and self-referential processing suggests a mode of brain function that can be optimized for clarity, non-reactivity, and present-centered awareness in daily life. This neural pattern directly inverts patterns observed in psychopathology, such as rumination in depression (59), hyper-reactivity in anxiety (60), and craving in addiction (61, 62). Instead of the disrupted prefrontal–limbic activity seen in these conditions, ACAM-J is characterized by lower prefrontal activity, higher posterior/midline activity, patterns that covaried with equanimity-related traits and were anticorrelated with psychological suffering. Equanimity has been proposed as a critical outcome for contemplative training and a contributor to stress resilience (37, 63–67), and advanced meditation such as ACAM-J and advanced investigative insight meditation explicitly cultivates deep absorption and equanimity compared to standard mindfulness programs (17, 68).

Moreover, cultivation of self-generated bliss and joy during early ACAM-J represents a transformative aspect of meditation practice with profound implications for mental health. Bliss, as experienced in ACAM-J, is not merely a fleeting positive emotional state but a self-generated, stable source of joy that emerges through absorption. The neural correlates of such ACAM-J suggest a shift away from self-referential processing towards a more unified, present-centered awareness that is richly positive and deeply fulfilling. This experience of joy, free from external stimulus, could fundamentally frameworks of psychiatric care by providing individuals with an internal reservoir of emotional well-being. Unlike conventional therapeutic models that focus on alleviating negative symptoms, practices like ACAM-J offer a means of fostering positive emotional states, potentially rewiring the brain to maintain these states even in the face of adversity.

In this context, advanced meditation could therefore provide a powerful naturalistic model of well-being and flourishing achieved through advanced mental training rather than pharmacological or technological interventions. Cultivation of intrinsic joy and bliss through advanced meditation may offer a novel pathway to alleviate cycles of rumination, self-reference, and emotional reactivity that characterize many psychiatric conditions. In essence, rather than merely reducing negative symptoms, ACAM-J could actively promote the cultivation of positive states, fostering emotional balance, and a profound sense of well-being. On this basis, we hypothesize that ACAM-J may result in stronger or qualitatively distinct well-being relative to standard mindfulness meditation, though direct comparative trials are lacking and are needed to test this prediction (15, 16).

Potential clinical applications of ACAM-J could include neuromodulations (69) to facilitate development of ACAM-J factors, or other advanced meditation states during meditative development (70). Techniques such as transcranial magnetic stimulation or focused ultrasound could be explored to target neural circuits associated with absorption, equanimity, and or self-generated bliss and happiness in clinical populations (71). Additionally, neurofeedback and closed-loop brain-computer interfaces may offer non-invasive methods to train and stabilize advanced meditation states. Ultimately, our study helps lay the groundwork for a rigorous science of advanced meditation, its states, development, endpoints, and flourishing, with profound implications for mental health and human well-being.

Notwithstanding its merits, this study has several limitations. First, even highly trained meditators may face challenges in reproducing deep absorption within the noisy MRI environment, and atypical supine position for practicing ACAM-J. Such ecological mismatch could alter phenomenology relative to naturalistic practice. However, we mitigated this by having participants practice ACAM-J with the MRI noise recording while laying supine prior to data collection. Moreover, the convergence across multiscale neural and experiential measures suggests that the core neural correlates of ACAM-J were reliably captured. The absence of electrophysiological recordings such as EEG or MEG limits characterization of fast spectral–temporal features such as microstates or non-linear oscillatory dynamics (26). The current findings may not generalize to other forms of absorption or concentration-based practices, which may differ in techniques, phenomenology, and neural signatures. Finally, deeper ACAM-J are increasingly subtle and inherently more difficult to sustain in the MRI system. While participants were able to enter these states, the requirement of button-press confirmation may perturb the absorption process. Unequal ACAM-J dwell times could also bias trajectory estimates. Nevertheless, participants, on average, maintained balanced durations across ACAM-J, mitigating this concern. Moreover, participants also indicated high quality of phenomenology of each ACAM-J and were able to indicate access to these eight states. Future research should incorporate multimodal recordings and longitudinal training designs to test and extend the results of the current study.

Our findings demonstrate that human consciousness is not fixed to its default modes but can be intentionally reconfigured into eight distinct absorption states marked by profound ease, stability, equanimity, and reduced ordinary perception and cognition. The eight distinct ACAM-J challenge prevailing neuroscientific models and theories by demonstrating that the brain harbors latent capacities capable of counteracting the maladaptive neural patterns that underlie rumination, craving, and hyper-reactivity. ACAM-J offers a novel experimental model of resilience, mental health, and flourishing, and demonstrates that the human brain carries within it the latent ability to catalyze its own transformative potential.

## Materials and Methods

### Participants

Participants were recruited as part of a larger study that aims to investigate neurophenomenology of various advanced meditation practices. These experienced meditators come from well-established meditation communities, all of whom demonstrated advanced expertise in ACAM-J meditation practices. The sample consisted of 20 participants, with an age range of 26–67 years old (mean age = 43.95 ± 15.10 years).

On average, participants had 17.40 ± 11.59 years of meditation experience and reported an estimated total of 20500.40 ± 17288.98 hours of dedicated meditation practice. Participants were further categorized based on their engagement with distinct styles of ACAM-J meditation, including intermediate (N=18), and deep (N=2) ACAM-J. The study received approval from the Mass General Brigham Institutional Review Board (IRB) and the ethics boards at Witten/Herdecke University. Participants also provided informed consent.

### Experimental design

#### Advanced concentrative absorption meditation (ACAM-J)

Participants followed their standard ACAM-J sequence with closed eyes, progressing from access concentration through ACAM-J1 to ACAM-J8, concluding with a post-ACAM-J8 state referred to as *afterglow*. We requested participants to stay within any ACAM-J for at least 3 minutes for reliable computation of fMRI metrics (72). For participants with ACAM-J duration of less than 2 minutes, we conducted another session of ACAM-J. The participants marked transitions from access concentration to ACAM-J8 with a button press. Note that 1 participant did not indicate transitions from ACAM-J6 to ACAM-J7 nor from ACAM-J7 to ACAM-J8, as it would disrupt the natural flow of ACAM-J practice for them. In total, 22 runs of ACAM-J were recorded, and the average duration for a complete run covering ACAM-J1 to ACAM-J8 was 33.60 ± 15.40 min (for more details on state duration, refer to Table S1).

#### Non-meditative control conditions

Two non-meditative control conditions were developed for comparison with meditation states, aimed at engaging the participant’s mind in non-meditative, ordinary cognitions while avoiding the induction of meditative states. Resting-state control was not used due to concerns that experienced meditators might enter meditative states during rest, which may complicate interpretations of results (73). These control conditions consider the phenomenology of meditative states and the nature of the experimental paradigm to ensure greater consistency and reliability in the intensive case-study design (74). The two control conditions involved a memory task, where the participant recalled events from the past one week, and a counting task, where the participant mentally counted down from 10,000 in decrements of 5. One 8 min runs of each non-meditative control were collected for most participants. Five participants who were recruited additionally for separate case studies conducted two sessions of the control tasks. In total, we collected 25 sessions of control tasks. Please note that some participants reported experiencing a small degree of ACAM-J during the control conditions. However, these meditative qualities were not as intense as the meditation states experienced during their meditation runs. Moreover, effects of ACAM-J during control conditions did not impact the differences between ACAM-J and non-meditative controls in prior analyses (74).

#### Phenomenology

To complement objective neuroimaging data, this study design also used a first-person phenomenological approach, in which the subject assessed the mental and physiological processes relevant to the experience of ACAM-J as they manifested during meditation. In brief, following a complete ACAM-J run (ACAM-J1 to ACAM-J8), the participant assigned a rating from 1 to 10 for phenomenological items including: (1) *stability of attention*, ranging from poor to excellent stability; (2) *width of attention*, varying from a narrow scope like a laser to very wide like a fisheye lens; (3) *quality of ACAM-J*, indicating the degree of alignment between experience of the ACAM-J quality and the defining features of the corresponding ACAM-J; (4) *early phenomenology of sights, sounds, physical sensations, and narrative thought stream*, denoted by the presence of sights, sounds, physical sensations, and narrative thought contents during the form ACAM-J (ACAM-J1-J4); (5) *late phenomenology of sights, sounds, physical sensations, and narrative thought stream*, characterized by the presence of sights, sounds, physical sensations, and narrative thought contents during the formless ACAM-J (ACAM-J5-J8); and (6) qualities of specific ACAM-J (ACAM-J factors) rated specifically for that ACAM-J, including *bliss/joy* (ACAM-J1–2), *contentment* (ACAM-J3), *equanimity* (ACAM-J4), and *formlessness* (ACAM-J5–8). Higher rating values indicated a more robust experience within the given item. *Stability of attention, width of attention,* and *quality of ACAM-J* were rated for each ACAM-J. Phenomenology of *sights, sounds, physical sensations, and narrative thought stream* were rated separately for early (ACAM-J1-J4) and late (ACAM-J5–8) aggregate states.

#### Questionnaires

Participants also completed several questionnaires regarding meditative traits as part of a larger study on advanced meditation. Here, we focused on questionnaires relevant to this study.

##### Five facet mindfulness questionnaire (FFMQ-15).

The FFMQ-15 is a shortened version of the original 39-item measure, assessing five distinct facets of mindfulness: observing, describing, acting with awareness, non-judging of inner experience, and non-reactivity to inner experience (75). Each facet is measured by three items rated on a 5-point Likert scale (1 = “never or very rarely true” to 5 = “very often or always true”).

##### Cognitive and Affective Mindfulness Scale- Revised (CAMS-R).

The CAMS-R is a 12-item self-report measure designed to capture mindfulness as a unified construct encompassing attention, awareness, present-focused acceptance, and non-judgment (76). Items are scored on a 4-point Likert scale (1 = “rarely/not at all” to 4 = “almost always”), with higher scores indicating greater mindfulness.

##### Non-attachment scale-short form (NAS-SF).

The NAS-SF measures the Buddhist psychological construct of non-attachment, defined as a flexible, balanced way of relating to one’s experiences without clinging to or suppressing them (77). The 8-item scale assesses the extent to which individuals can engage in life with less fixation on outcomes and experiences. Items are rated on a 6-point Likert scale (1 = “strongly disagree” to 6 = “strongly agree”), with higher scores indicating greater non-attachment.

##### Equanimity scale (ES-16).

The ES-16 measures the ability to maintain mental balance regardless of whether experiences are pleasant, unpleasant, or neutral (78). This 16-item self-report measure captures two subscales: experiential acceptance, an attitude which does not seek to resist or attach to the experience and involves acceptance of all internal experiences (thoughts, feelings, body sensations, etc.), and non-reactivity, which is non-reactivity to experiences preventing attachment or aversion to these experiences (e.g. thoughts, feelings). Items are rated on a 5-point Likert scale (1 = “not at all” to 5 = “very much”), assessing even-minded states and non-preferential reactivity to experiences.

##### Attentional Control Scale (ACS).

The ACS is a 20-item scale assessing attentional control, focusing on the ability to focus, shift attention between tasks, and flexibly control thought (79). ACS has two subscales: attentional focusing, which is the capacity to intentionally hold the attentional focus, resisting unintentional shifting to irrelevant or distracting stimuli, and attentional shifting, which is the capacity to intentionally shift the attentional focus to the desired target, avoiding unintentional attention. Items are rated on a 4-point Likert scale (1 = “almost never” to 4 = “always”), with higher scores indicating better attentional control.

#### Cognitive tasks

We examined six 16 standardized tasks assessing various cognitive domains using TestMyBrain (TMB) Digital Neuropsychology Toolkit (80). Normative data for the TMB battery, collected from samples of 4000–60,000 individuals aged 12–90 years, are stratified by age, gender, level of education, and type of device used. Specific tests include domains of processing speed: Simple Reaction Time (SRT; measure of basic processing speed), Choice Reaction Task (CRT; measure of decision-making speed with multiple stimuli), Digit Symbol Matching (DSST; measure of processing speed), Trail Making Tests Part A (TMT_A; measure of processing speed); cognitive control: Trail Making Tests Part B (TMT_B; measure of cognitive flexibility), Gradual Onset Continuous Performance Test (CPT; measure of sustained attention and vigilance); working memory: Paced Serial Addition Test (ADD; measure of sustained attention and working memory), Forward and Backward Digit Spans (FORW and BACK_SPAN; measure of working memory), Multiple Object Tracking (OBJ_TRACK; measure of visuospatial attention and visual working memory); reasoning: Matrix Reasoning (MATRIX_REASON; measure of visual reasoning and general cognitive ability); long-term memory: Verbal and Visual Paired Associates Memory Tests (VERBAL_MEM and VISUAL_MEM; measures of verbal and visual memory, respectively), Cambridge Face Memory Test (CAMFACE; measure of face recognition), and social function: Multiracial Emotion Identification (EMO_IDEN; measure of face emotion recognition), and Reading the Mind in the Eyes (READ_MIND_EYES; measure of mental state inferencing and theory of mind). For more information about these measures and the domains that they measure, please refer to Supplementary Table 1.

### Neuroimaging acquisition

Neuroimaging scans were obtained utilizing a 7T magnetic resonance (MR) scanner (SIEMENS MAGNETOM Terra) equipped with a 32-channel head coil. Functional imaging utilized a single-shot two-dimensional echo planar imaging sequence with T2*-weighted BOLD-sensitive MRI. The imaging parameters included a repetition time (TR) of 2.9 s, echo time (TE) of 30 ms, flip angle (FA) of 75 degrees, field of view (FOV) of [189 × 255], matrix of [172 × 232], GRAPPA factor of 3, voxel size of 1.1 × 1.1 × 1.1 mm^3^, 126 slices, interslice distance of 0 mm, bandwidth of 1540 Hz/px, and echo spacing of 0.75 ms. Slice acquisitions covered the entire brain, with interleaved slices, sagittal orientation, and anterior-to-posterior phase encoding. Additionally, opposite phase-encoded slices (i.e., posterior-to-anterior) with identical parameters were acquired for distortion correction.

Whole-brain T1-weighted structural images were acquired with the following parameters: TR of 2.53 s, TE of 1.65 ms, inversion time of 1.1 s, flip angle of 7 degrees, 0.8 mm isotropic resolution, FOV of 240 × 240, GRAPPA factor of 2, and bandwidth of 1200 Hz/Px. Physiological signals, including heart rate and respiration, were continuously monitored throughout the scanning session. Heart rate was measured using a piezoelectric finger pulse sensor (ADInstruments, Colorado Springs, CO, USA), and respiration was recorded using a piezoelectric respiratory bellow (UFI, Morro Bay, CA, USA) positioned around the chest. The signals were acquired using a PowerLab data acquisition system (PowerLab 4/SP, ADInstruments, New Zealand) and LabChart (LabChart, ADInstruments, New Zealand) at a sampling rate of 1000 Hz. The same acquisition system also recorded MR scanner trigger pulses synchronized with the acquisition of each image volume.

### Neuroimaging preprocessing

Detailed processing steps can be found in a previous case study (14). In brief, preprocessing pipeline encompassed the following steps: 1) de-spiking, 2) RETROspective Image CORrection (RETROICOR), 3) slice time correction, 4) distortion correction, 5) motion correction, 6) registration of the anatomical dataset (T1w) to a standard MNI template, 7) scrubbing and removal of any volume with motion >0.3 mm and more than 5% outlier voxels, 8) regression of eroded cerebrospinal fluid (CSF) mask time course and motion parameters (3 translations, 3 rotations) per run, and finally band-pass filtering (0.01–0.1 Hz). Each fMRI run was then partitioned into distinct segments corresponding to different ACAM-J. Finally, 9) the bias field of the T1 image was corrected using SPM12 (82).

### Regional homogeneity

Regional homogeneity (ReHo) is a measure of similarity in temporal activation pattern of a voxel and its nearby voxels (83). This measure of local functional connectivity within brain regions is a close derivative of underlying brain activity (83). A higher ReHo value indicates stronger synchronization of local brain regions, indicating greater functional connectivity in that region and thus an index of greater brain activity. In this study, we defined a cluster size of 27 and calculated the ReHo values for each fMRI segment, standardized the ReHo values before smoothing the standardized ReHo maps using a 2mm full-width half-maximum (FWHM) kernel. The standardized ReHo values were then parcellated using the four different parcellation/segmentation schemes (84–87), yielding 498 ReHo values for each fMRI segment.

### Connectivity gradient-mapping

To map the gradients, we first computed a template gradient through a multi-step process. First, data was smoothed using a 6 mm FWHM kernel, followed by computation of functional connectivity matrices for each segment using the Schaefer 400-regions 7-network atlas using Pearson’s correlation, thus producing a 400×400 functional connectivity matrix. These individual matrices were then averaged across all segments, resulting in a mean functional connectivity matrix. Subsequently, gradients for this mean matrix were derived by thresholding the mean correlation matrix at 90% sparsity to retain only the strongest connections, followed by generating a similarity matrix using cosine similarity to capture the connectivity pattern similarities among ROIs. The resulting similarity matrix served as input for the diffusion map embedding algorithm, from which the gradients were extracted. We computed a template gradient from an out-of-sample dataset of 134 subjects from the HCP dataset (the validation cohort used by (88)).

Following template gradient computation, alignment with individual segment gradients was performed. The gradient values for each fMRI segment were computed based on their respective functional connectivity matrices and then aligned with the template gradient using Procrustes rotations with 10 iterations to ensure optimal comparison across datasets. All analyses described herein were conducted using the *BrainSpace* toolbox implemented in Python (88). Using the gradient values obtained from gradient alignment, we computed the average network gradient values for each network by averaging the gradient values of all the parcels within that network. To ease interpretation and reduce the complexity of the current manuscript, we focused only on the principal gradients (G1). An extended analysis of the connectivity gradients may be conducted in future studies.

### Geometric eigenmode decomposition

All fMRI data was smoothed with surface-smoothing of 6 mm FWHM, and was converted to CIFTI data format for further processing *using ciftify_recon_all* and *ciftify_subject_fmr i*(89). To decompose fMRI activity into 200 frequency-specific eigenmodes, we used geometric eigenmodes derived from a population-averaged midsurface thickness template of the neocortical surface from Pang et al., wherein the methods to generate these geometric eigenmode templates are also described (45). Briefly, the geometric eigenmodes were computed by constructing the Laplace-Beltrami operator from the cortical mesh and solving the eigenvalue problem. The resulting eigenvalues are ordered according to spatial frequency/wavelength of each mode, where mode 1 has the longest wavelength (lowest frequency) while higher modes have shorter wavelengths (higher frequency). Conversely, eigenmodes are the complete basis set—a weighted sum of varying-wavelength modes.

Using the geometric eigenmodes, we decomposed functional MRI data to reveal emerging cortical spatiotemporal dynamic patterns by examining the contribution of each eigenmode to the cortical activity at each time instance. At each time point of each fMRI time courses, fMRI data were projected onto each of the 200 geometric eigenmodes, yielding the temporal activity of the particular eigenmode in a method described previously (44, 90). Subsequently, the temporal activation of eigenmodes were analyzed in terms of their power and energy at each fMRI time point. Here, power is defined as the strength of an eigenmode’s activation at a given fMRI time instance: |ω_k_(t_i_)|. Alternatively, energy is the eigenmode’s frequency-weighted contribution, estimated by weighting the square of the eigenmode’s strength of activation by the square of its corresponding eigenvalue (i.e. its intrinsic energy (*λ*_*k*_)) at each time point: |ω_k_(t_i_)|^2^ *λ*_*k*_^*2*^. Mean power and energy were calculated by averaging the power and energy of each eigenmode across all time points in a single segment (i.e. yielding 200 mean power and energy values for 200 modes per fMRI segment). Maximum power and energy were calculated as the maximum spectra value for a given eigenmode across all time points within a segment (i.e. yielding 200 maximum power and energy values for 200 modes per fMRI segment). Unless otherwise noted, all reported maximum and mean power and energy values are reported on a log-scale (44). Brain total power and total energy were computed by summing power and energy across all eigenmodes for each time point and then averaging all time points within a segment. We focus on global mean power and energy patterns, trends, and frequency-specific mean power and energy across ACAM-J in the main results. We present the same analyses, but for max power and energy patterns in the supplementary results.

### Decoding of brain activity using data-driven Neurosynth psychobehavioral affinity maps

To provide a complementary approach for analyzing brain activity patterns with behavior in the absence of behavioral data, we examined the spatial similarity of ReHo maps with 123 meta-analytic brain maps derived from the Neurosynth database. Neurosynth generates probabilistic measurements that can be interpreted as a quantitative representation of the association between regional fluctuations in activity and psychological processes. Although Neurosynth’s database contains over 1,000 terms, we narrowed our focus to terms related to mental processes and behavior, drawing from previous research (91–93). Our selected set of 123 terms encompasses broad categories, ranging from attention and emotion to more specific processes such as visual attention and episodic memory. The terms also include basic behaviors (e.g., eating and sleep) and emotional states (e.g., fear and anxiety).

### Statistical analysis

For participants with two sessions of neuroimaging sessions (either ACAM-J or control), metrics were averaged across sessions to reduce within-subject variability and simplify statistical modeling. Condition-level comparisons focused on a predefined mask derived from regions previously shown to be sensitive to ACAM-J effects in our earlier case studies. This targeted approach increased statistical power by restricting analyses to brain areas with known relevance. Complementary whole-brain analyses are reported in the Supplementary results. Missing phenomenology, and meditative traits were imputed using multivariate imputation by chained equations (MICE). This method was selected due to its robustness in preserving the multivariate relationships among variables, and its ability to produce unbiased parameter estimates under the assumption of missing at random (MAR) (94, 95).

#### Trends in neuroimaging metrics across ACAM-J

Linear mixed models (LMM) was used to examine polynomial trends up to the 3^rd^ degree (cubic trend) across ACAM-J for each neuroimaging metric. We implemented orthogonal polynomial contrast coding to test for linear, quadratic, and cubic relationships between the neuroimaging metric and the progression through ACAM-J. This approach allows us to capture potential non-linear patterns that might emerge across the ACAM-J progression, as shown in our previous case studies (14, 25, 27, 68). The polynomial terms were centered to reduce multicollinearity. In models with non-significant higher-order trends (e.g., non-significant cubic trend), we reduced the polynomial term iteratively (e.g. including quadratic and linear, or linear trend only) to simplify the model to best characterize the relationship between ACAM-J progression and the neuroimaging metric. Participant was also included as a random intercept individual differences in baseline neuroimaging metrics. Significance of models and polynomial terms were corrected for multiple comparisons using false discovery rate (FDR)–adjusted q-values. Models were corrected for multiple comparisons using FDR across the number of parcellations within each neuroimaging metric. Additional details for each neuroimaging metric are presented in subsequent sections. Analyses were conducted in *R* (96) loading on R Studio v2022.12.0.353.20 (97).

#### Comparisons between ACAM-J and non-meditative control conditions

We first assessed whether the two non-meditative control conditions (counting and memory) differed significantly. To do so, we fit LMMs with control condition as the predictor and participant as the random intercept. Pairwise contrasts between counting and memory were computed, with FDR correction applied across the models to control for multiple testing.

Unless stated otherwise, using this composite control as the reference level, we then applied LMM to compare ACAM-J against the control condition. Each model included ACAM-J as a categorical predictor and participant as the random intercept. Estimated marginal means and pairwise contrasts were computed for each ACAM-J versus control comparison. Contrast coefficients within each model were corrected for multiple comparisons using FDR, and an additional FDR correction was applied across all models to control for multiple testing.

We also conducted supplementary analyses comparing the same neuroimaging metrics between ACAM-J and the control conditions separately to ensure robustness in our analysis. Results for this set of analysis are reported in the supplementary materials.

#### Multivariate brain-behavior relationships: Linking neuroimaging metrics with phenomenology, meditative traits, and cognition

Unless stated otherwise, we used partial least squares correlation (PLSC) analysis to investigate the relationships between neuroimaging metrics and three domains of behavioral measures: phenomenology, meditative traits, and cognition. PLSC is a multivariate technique that identifies latent variables (LVs) representing patterns of covariance between brain activity and behavioral data (98). Phenomenological experience during ACAM-J and non-meditative tasks include quality of ACAM-J (none for control tasks), stability of attention, width of attention, sensation of sights, sounds, physical sensations, and narrative thought stream. Measures of meditative traits include the five dimensions of FFMQ (observing, describing, acting with awareness, non-judging of inner experience, and non-reactivity to inner experience), CAMS-R, NAS-SF, ES-16, two subscales of ACS (attentional focusing and attentional shifting), and IAI. Cognitive performance include all the 16 cognitive tasks as described previously.

For each analysis, we specified the number of components equal to the number of behavioral variables in the respective domain. Statistical significance of each latent variable was assessed using a 10,000 permutations to generate null distributions of singular values (99), with p_FDR_ < 0.05 considered significant (100–102). Bootstrap resampling with 10,000 iterations was used to estimate the reliability of brain and behavioral weights. The Kaiser criterion was applied to determine component significance, retaining components that explained more variance than the average across all components. Pearson’s correlations were conducted to examine the linear relationship between the latent X and Y variables.

For ReHo and principal connectivity gradients, PLSC was conducted at the ROI-level. For ReHo, we conducted PLSC using 498 ReHo ROIs, while PLSC was conducted using 400 cortical gradients ROIs for connectivity gradients. For geometric eigenmodes, we used global eigenmode activations (mean/max power/energy).

#### Univariate brain-behavior relationships linking neuroimaging metrics with specific ACAM-J factors.

Given that each ACAM-J has specific qualities, we also conducted Pearson’s correlation between the neuroimaging metrics and ACAM-J factors. In total, four sets of correlations were conducted within each neuroimaging metric: Bliss/joy (ACAM-J1–2), contentment (ACAM-J3), equanimity (ACAM-J4), and formlessness (ACAM-J5). Analyses were corrected for multiple testing with FDR within the specific set of analysis, i.e., within all ROIs for each ACAM-J factor.

#### Robustness Checks: Subgroup Analyses

To assess the robustness of our primary findings, we conducted a series of subgroup analyses designed to account for potential heterogeneity in meditative experience and style. Specifically, we performed a secondary analysis excluding two deep-level ACAM-J practitioners (resulting in N = 18), as different forms or depths of ACAM-J practice may be associated with distinct phenomenological and neurobiological profiles. This exclusion aimed to isolate the effects of intermediate-level practice and reduce potential confounds introduced by advanced practitioners. Full statistical results and neuroimaging data for this subgroup analysis are reported in the supplementary materials.

Additionally, we evaluated the spatial correspondence of neural activation patterns between two subtypes of intermediate ACAM-J practice: Intermediate-Sutta-style and Intermediate-Tranquil Wisdom Insight Meditation (TWIM). To do this, we performed a permutation-based correlation analysis. First, we generated significant delta-ReHo maps for two subgroups: Subgroup A, which included all participants (N = 20), and Subgroup B, which excluded three TWIM-style ACAM-J practitioners (N = 3). We then computed the correlations of ReHo values across all ACAM-J between these two subgroups.

To assess the statistical robustness of the observed correlations, we conducted a permutation test with 10,000 iterations. This analysis allowed us to determine whether the inclusion or exclusion of TWIM-style practitioners significantly influenced the observed ReHo patterns. A high degree of correlation would indicate that TWIM and Sutta-style ACAM-J practices produce comparable neural signatures, suggesting a shared underlying phenomenological structure.

Finally, we conducted a simple linear regression analysis to examine whether spatial similarity between the two subgroups increased progressively across ACAM-J, thereby evaluating the stability and convergence of the neural signatures over ACAM-J.

#### Contextualization of brain activity with Neurosynth psychobehavioral affinity maps

We used the *continuous.CorrelationDecoder* function from the NiMARE python package to contextualization of brain activity with Neurosynth psychobehavioral affinity maps (103). We first fetched Neurosynth dataset and converted it to NiMARE dataset. Following, we retrieved the 123 psychobehavioral terms from the database. We also computed delta-ReHo maps for every ACAM-J pair with the control composite (e.g., ACAM-J1-control, ACAM-J2-control) for decoding. Finally, we used the *CorrelationDecoder* function to decode the association between the unthresholded delta-ReHo maps and psychobehavioral affinity maps.

## Supplementary Material

Supplement 1

## Data Availability

Data may be requested and is subjected to the Massachusetts General Hospital Institutional Review Board’s guidelines and approval. All code used in this study may be made available by request from the corresponding contact email address.
