# Supplementary material for: The neuroscience of highly stable, positive, and refined states of consciousness during jhana-type advanced concentration absorption meditation (ACAM-J)
Source: bioRxiv. 2025 Nov 13:2025.11.12.688050. Preprint. [Version 1] doi: 10.1101/2025.11.12.688050 (PMC12642441; doi:10.1101/2025.11.12.688050)
Supplement: Supplement 1 [file NIHPP2025.11.12.688050v1-supplement-1.pdf]

bioRxiv preprint doi: https://doi.org/10.1101/2025.11.12.688050; this version posted November 13, 2025. The copyright holder for this preprint (which was not certified by peer review) is the author/funder, who has granted bioRxiv a license to display the preprint in perpetuity. It is made available under aCC-BY 4.0 International license.

Supplementary Materials

Supplementary Text

Dominant phenomenology of each ACAM-J

ACAM-J1. The dominant phenomenology of ACAM-J1 is one of joyful seclusion based on directed and sustained attention. Upon entering this state, the meditator experiences a distinct withdrawal from external sensations, internal monologue and narrative thoughts. The mind, with high stability and unification, is directed towards the meditation object with which it also experiences some degree of merger or absorption. This unified state is accompanied by five primary ACAM-J factors: directed attention, sustained attention, bliss, happiness, and one-pointedness of the mind. The most prominent feelings are the emergence of bliss and happiness. Bliss is an energetic, often physical joy, like thrilling waves, while happiness is a more serene, pervasive happiness. The meditator may still need to actively focus on the object and adjust the balance between effort and relaxation, so there is a quality of effortful engagement, but the overwhelming experience is seclusion, relief, delight, and the novel stability of a mind free from its usual mode of perception, planning, and thinking.

ACAM-J2. The dominant phenomenology of ACAM-J2 is bliss, which may be experienced as goosebumps, waves, or pervasive, intense pleasure. Directed attention, sustained attention falls away, as the mind no longer needs to actively fixate on the meditation object and adjust the balance between relaxation and effort. The mind now rests there immovably on its own to a much stronger degree. Bliss and happiness that emerges from concentration intensify and become the most prominent phenomenology. Bliss can feel more powerful and pervasive than in ACAM-J1, while the underlying happiness deepens into a profound contentment.

ACAM-J3. The dominant phenomenology of ACAM-J3 is mindful equanimity together with a deep happiness. The energetic and sometimes overwhelming waves of bliss have faded away entirely, seen now as a subtle distraction. Happiness that seems to permeate the entire body remains. It is a state of contentment and fulfilling happiness. The primary phenomenology is a gentle and refined, all-pervading physical and mental pleasure coupled with an unshakable mental balance.

ACAM-J4. The dominant phenomenology of ACAM-J4 is absolute neutrality. The subtle and refined happiness of ACAM-J3 is relinquished, along with any lingering trace of pain or dissatisfaction. The experience is defined by pure equanimity and one-pointedness. The mind is extremely still and unperturbed by internal or external stimulus. Consciousness is exceptionally bright and clear. ACAM-J4 serves as the foundation for the development of deep insight.

ACAM-J5. The dominant phenomenology of ACAM-J5 is the perception of boundless, limitless space. Attending to the concept “infinite space,” sets the mind free from all boundaries of location and physicality. The experience is one of profound expansion. The sense of bodily boundary and a physical space dissolves, replaced by a direct perception of space as an infinite, open field close to infinite awareness.

ACAM-J6. The dominant phenomenology is the direct experience of infinite and boundless consciousness. The object of meditation becomes the knowing quality of the mind itself, which is now seen to be as limitless as the space being perceived in ACAM-J5. The duality between the observer and the observed becomes blurred. It is a state of infinite field of knowing, aware of its

bioRxiv preprint doi: https://doi.org/10.1101/2025.11.12.688050; this version posted November 13, 2025. The copyright holder for this preprint (which was not certified by peer review) is the author/funder, who has granted bioRxiv a license to display the preprint in perpetuity. It is made available under aCC-BY 4.0 International license.

own unbounded nature. The primary experience is one of luminous, self-aware knowing that has no center and no periphery.

ACAM-J7. The dominant phenomenology of ACAM-J7 is the perception of "nothingness" in which the mind let go of the object of infinite consciousness and takes "nothingness" as its focus. This is not a state of unconsciousness or a blank void, but a highly refined perception of absence where something could have been present. It is the most subtle form of object-based perception, where the mind clings to the very last concept of "something", and in this case, the concept of "nothing".

ACAM-J8. The dominant phenomenology of ACAM-J8 is so subtle that it is difficult to describe conceptually with words. The mind enters a state where consciousness cannot be said to be perceiving something, and yet it can also not be said to be perceiving a minimal something, like an absence. The mental factors are operating at their absolute minimal level. The mind is not cognizing any object, even the most refined, yet it is not unconscious. It is a state of residual mental formation, operating on the very edge of cessation.

Differing depths of ACAM-J and subgroup analyses.

Different meditation traditions and schools have developed distinct interpretations and training methods for ACAM-J (*1*-*5*). Recent comparative work highlights three broad types of ACAM-J practice: light, intermediate, and deep (*1*). Intermediate ACAM-J emphasizes the cultivation of jhāna factors such as sustained effortless focus, joy, ease, and one-pointedness (*3, 6, 7*). These forms of ACAM-J are typically phenomenologically varied, permitting some thought-like activity, bodily awareness, and a more accessible entry into absorption. Teachers such as Leigh Brasington, Ayya Khema, and Bhante U Vimalaramsi represent this style. In contrast, light ACAM-J consists of less stable and more effortful focus, presence of narrative thought, and less pronounced presence of the ACAM-J factors both in terms of range and intensity. Deep ACAM-J, often associated with the *Visuddhimagga* (Path of purification in the Pali language) and teachers such as Pa Auk Sayadaw, is defined by absorption into a mental image or *nimitta* in the Pali language (*8, 9*). This form is phenomenologically stricter and entails very deep concentration in which bodily and sensory experience disappear entirely. Intermediate ACAM-J and deep ACAM-J differ most in absorption depth, the role of nimitta, and the extent of pacification of sensory input.

In our main analyses, we focus on the full sample of practitioners spanning these traditions (N=20), performing mostly intermediate ACAM-J, with a few cases of deep ACAM-J (N=2). We did not recruit ant light ACAM-J practitioners for this study. To assess robustness, however, we conducted a subgroup analysis which only includes intermediate ACAM-J (N=18). For the subgroup analysis, all control conditions were combined into a composite control, given minimal differences across them.

bioRxiv preprint doi: https://doi.org/10.1101/2025.11.12.688050; this version posted November 13, 2025. The copyright holder for this preprint (which was not certified by peer review) is the author/funder, who has granted bioRxiv a license to display the preprint in perpetuity. It is made available under aCC-BY 4.0 International license.

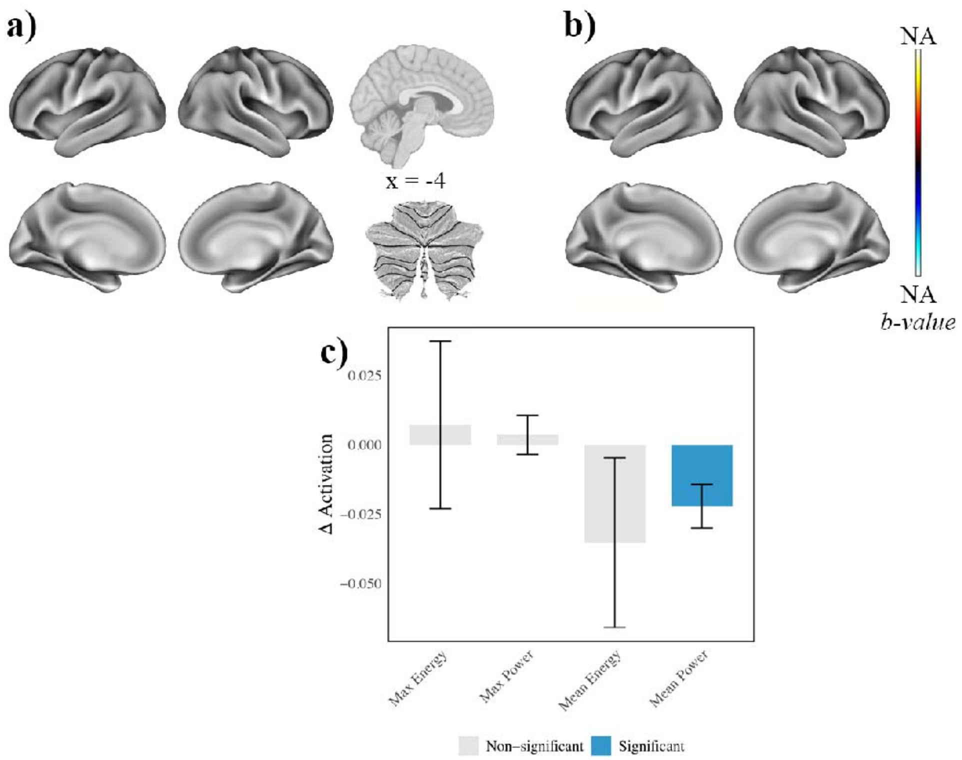

Figure S1. Comparison between memory and counting condition. (a-b) No significant differences in (a) ReHo and (b) G1 values were found between the two control conditions. (c) Mean power for the memory control condition was lower than the counting condition. Error bars represent standard errors.

bioRxiv preprint doi: https://doi.org/10.1101/2025.11.12.688050; this version posted November 13, 2025. The copyright holder for this preprint (which was not certified by peer review) is the author/funder, who has granted bioRxiv a license to display the preprint in perpetuity. It is made available under aCC-BY 4.0 International license.

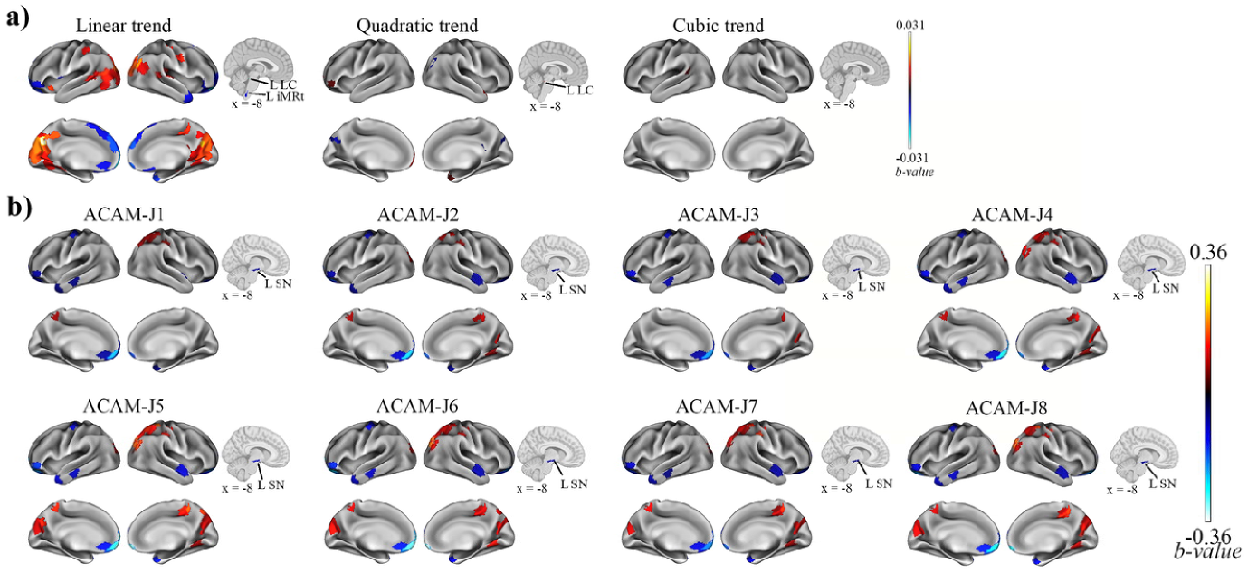

Figure S2. ReHo differences for ACAM-J vs the composite control condition (N=20) with multiple corrections across the entire brain (k = 498 ROIs). Trends analyses show regions exhibiting linear and quadratic trends across task ACAM-J. Linear trends highlights progressive and linear increase or decrease in specific brain regions while quadratic trends show mostly negative (inverted U-shaped) changes across ACAM-J. (b) ReHo differences between ACAM-J and the composite control reveal distinct patterns of cortical and brainstem substantia nigra activity.

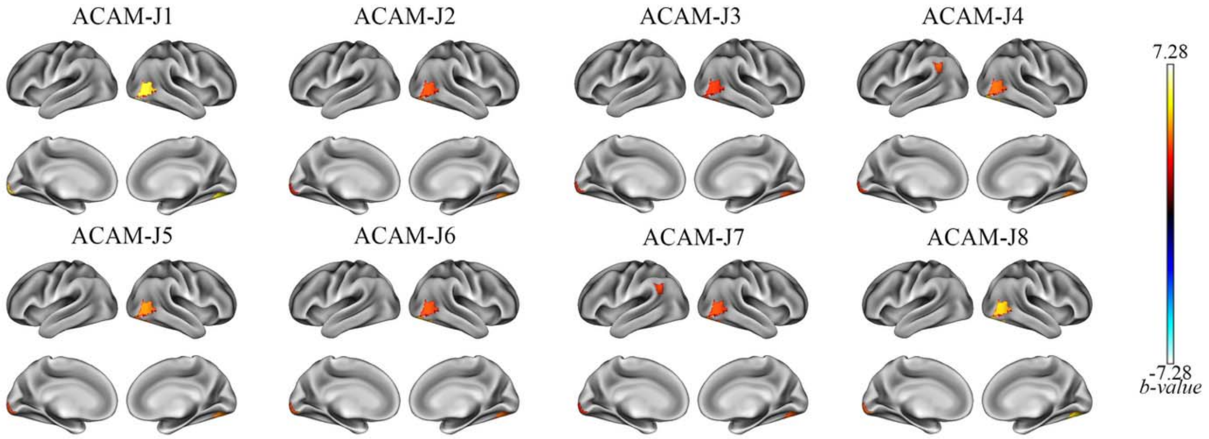

Figure S3. G1 differences for ACAM-J vs the composite control condition (N=20) with multiple corrections across the entire brain (k = 498 ROIs). No significant trends were found within ACAM-J. Contrasts between individual ACAM-J and the composite of control conditions reveal robust changes along G1 in visual and temporal hubs highlighting regional-specific reorganization of large-scale cortical hierarchy during ACAM-J.

bioRxiv preprint doi: https://doi.org/10.1101/2025.11.12.688050; this version posted November 13, 2025. The copyright holder for this preprint (which was not certified by peer review) is the author/funder, who has granted bioRxiv a license to display the preprint in perpetuity. It is made available under aCC-BY 4.0 International license.

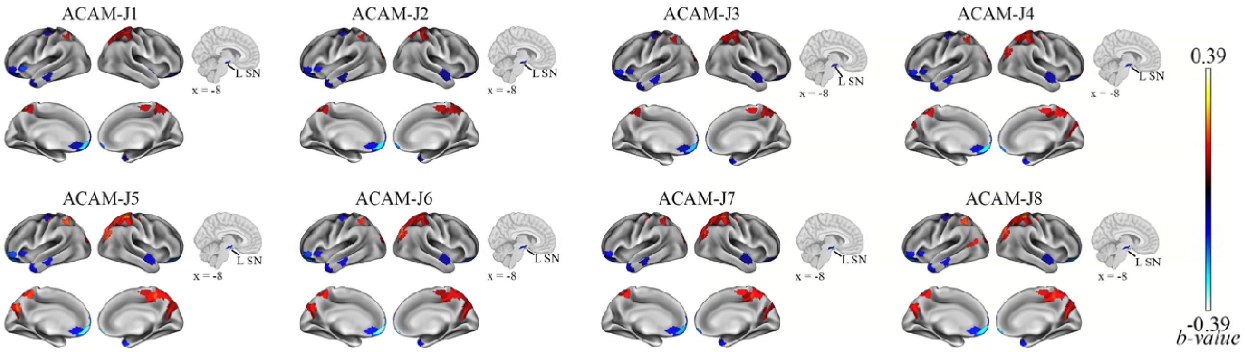

Figure S4. ReHo differences for ACAM-J vs counting condition in the full sample (N=20). Most ACAM-J were characterized by higher ReHo in the right posterior and somatomotor cortex and lower ReHo values in the bilateral orbitofrontal cortex (OFC), prefrontal cortex (PFC), and left substantia nigra. Later ACAM-J (ACAM-J5-8) had higher ReHo in the visual cortex.

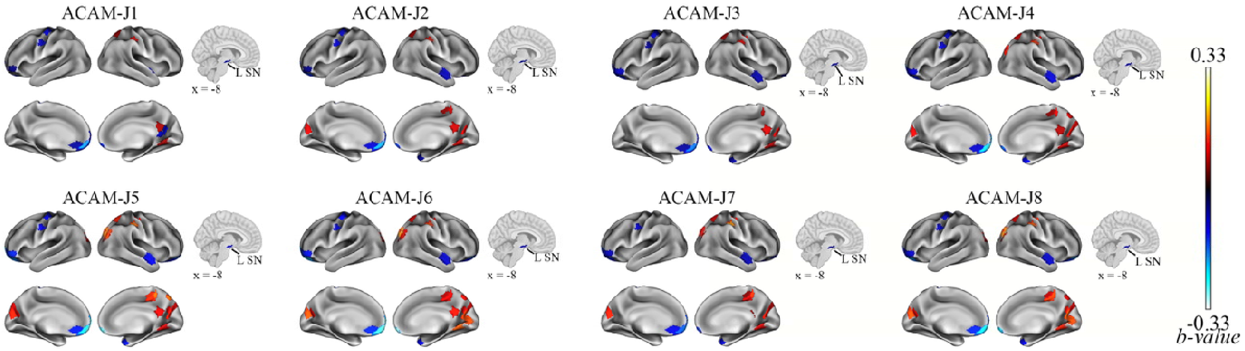

Figure S5. ReHo differences for ACAM-J vs memory condition in the full sample (N=20). Similar to **Figure S4**, most ACAM-J were characterized by higher ReHo in the right posterior and somatomotor cortex and lower ReHo values in the bilateral orbitofrontal cortex (OFC), prefrontal cortex (PFC), and left substantia nigra.

bioRxiv preprint doi: https://doi.org/10.1101/2025.11.12.688050; this version posted November 13, 2025. The copyright holder for this preprint (which was not certified by peer review) is the author/funder, who has granted bioRxiv a license to display the preprint in perpetuity. It is made available under aCC-BY 4.0 International license.

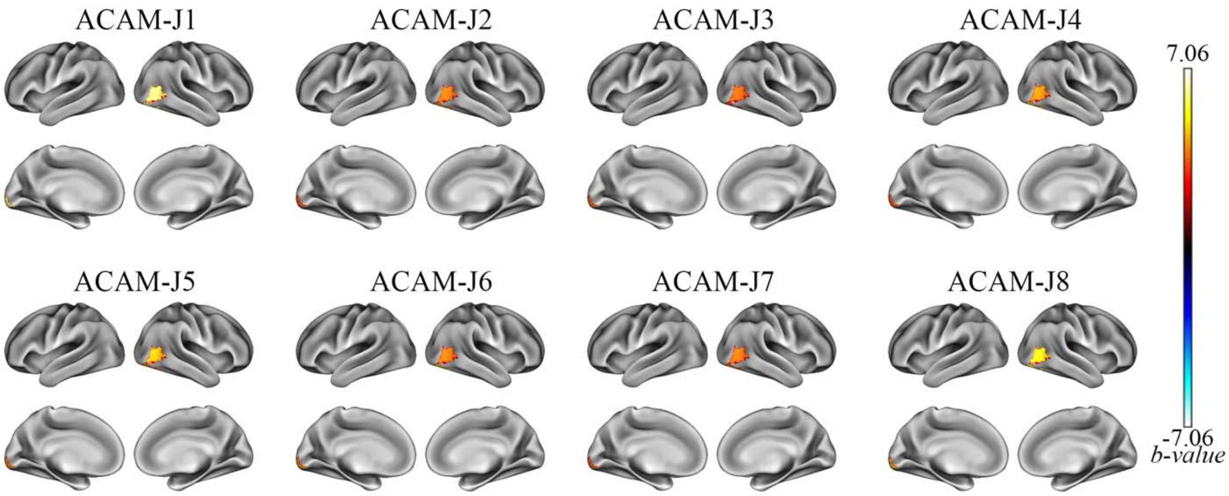

Figure S6. G1 differences for ACAM-J vs counting in the full sample (N=20). Relative to counting, most ACAM-J were characterized by higher G1 values in the visual cortex.

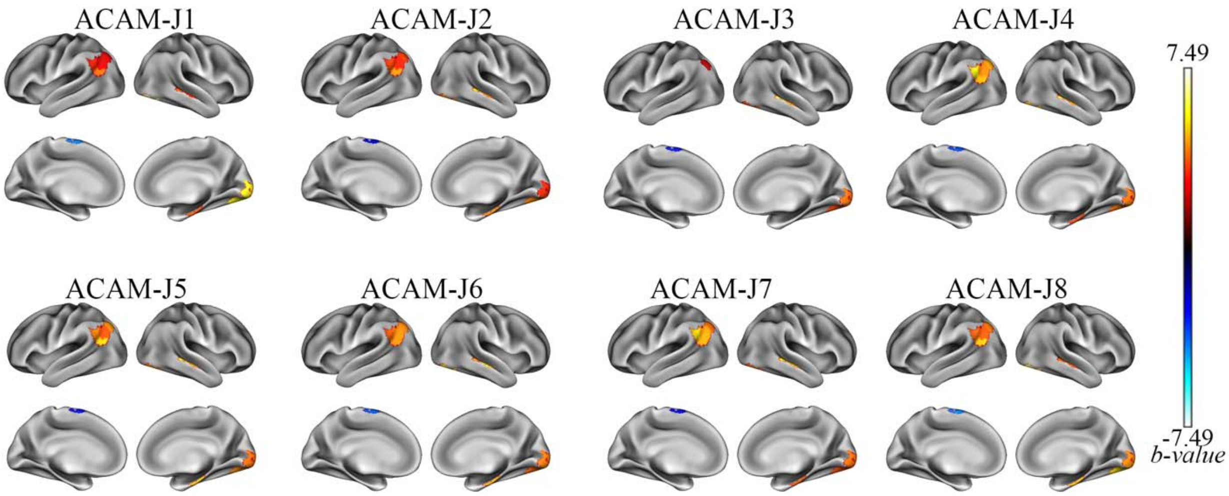

Figure S7. G1 differences for ACAM-J vs memory in the full sample (N=20). Compared to the memory control condition, most ACAM-J were characterized by higher G1 values in the visual cortex.

bioRxiv preprint doi: https://doi.org/10.1101/2025.11.12.688050; this version posted November 13, 2025. The copyright holder for this preprint (which was not certified by peer review) is the author/funder, who has granted bioRxiv a license to display the preprint in perpetuity. It is made available under aCC-BY 4.0 International license.

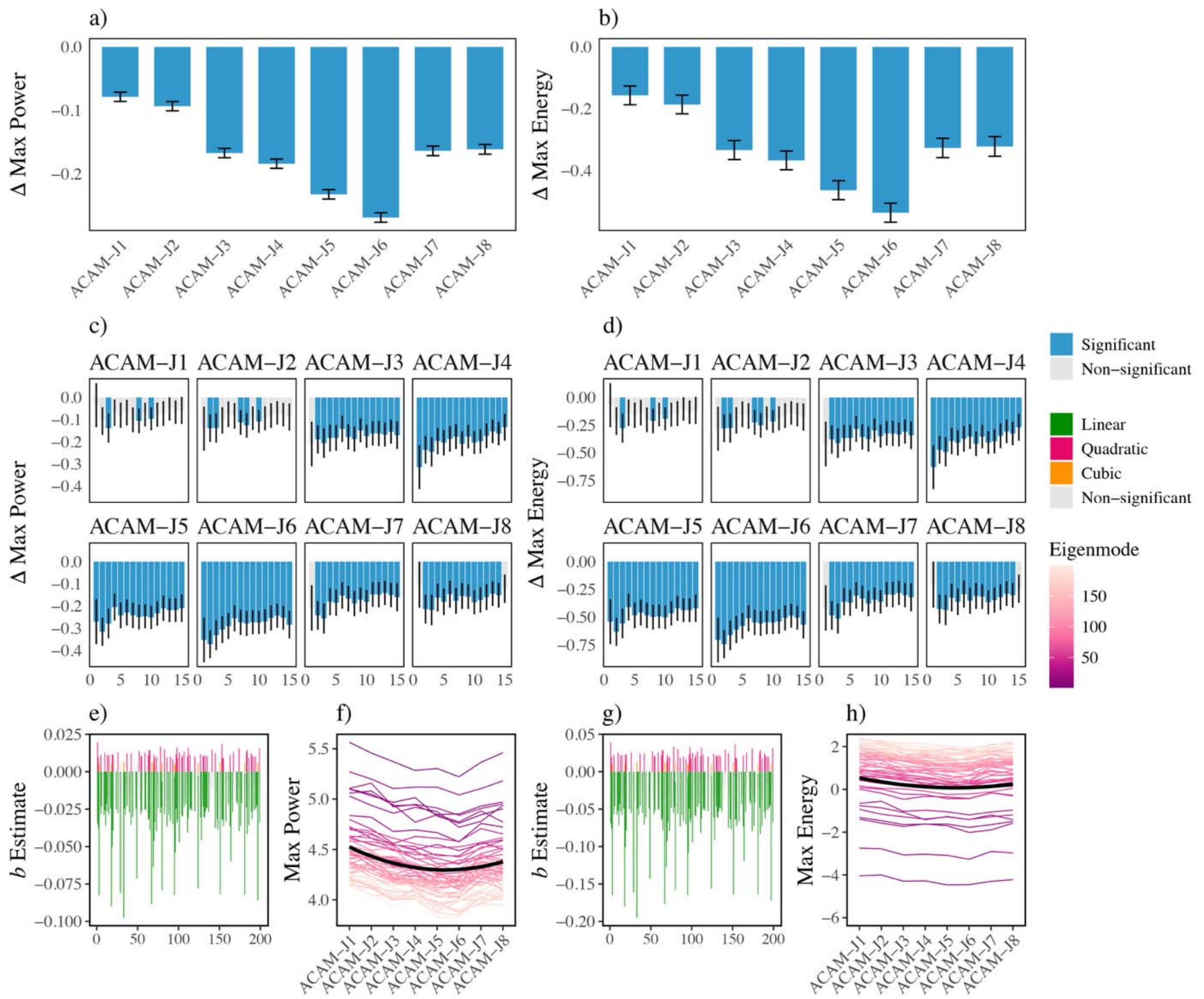

Figure S8. Global cortical dynamics in the full sample (N=20). (a-b) Relative to the control conditions, all ACAM-J showed lower max power/energy. (c-d) There were eigengroup nuances, with minimal significant differences for ACAM-J1 and ACAM-J2, and differences in most of the eigengroups from ACAM-J3 to ACAM-J8. (e-h) Both max power/energy exhibited similar negative linear and positive quadratic trends with mean power/energy, indicating an early decline from ACAM-J1 through ACAM-J5 followed by recovery in later ACAM-J. Error bars represent standard errors.

bioRxiv preprint doi: https://doi.org/10.1101/2025.11.12.688050; this version posted November 13, 2025. The copyright holder for this preprint (which was not certified by peer review) is the author/funder, who has granted bioRxiv a license to display the preprint in perpetuity. It is made available under aCC-BY 4.0 International license.

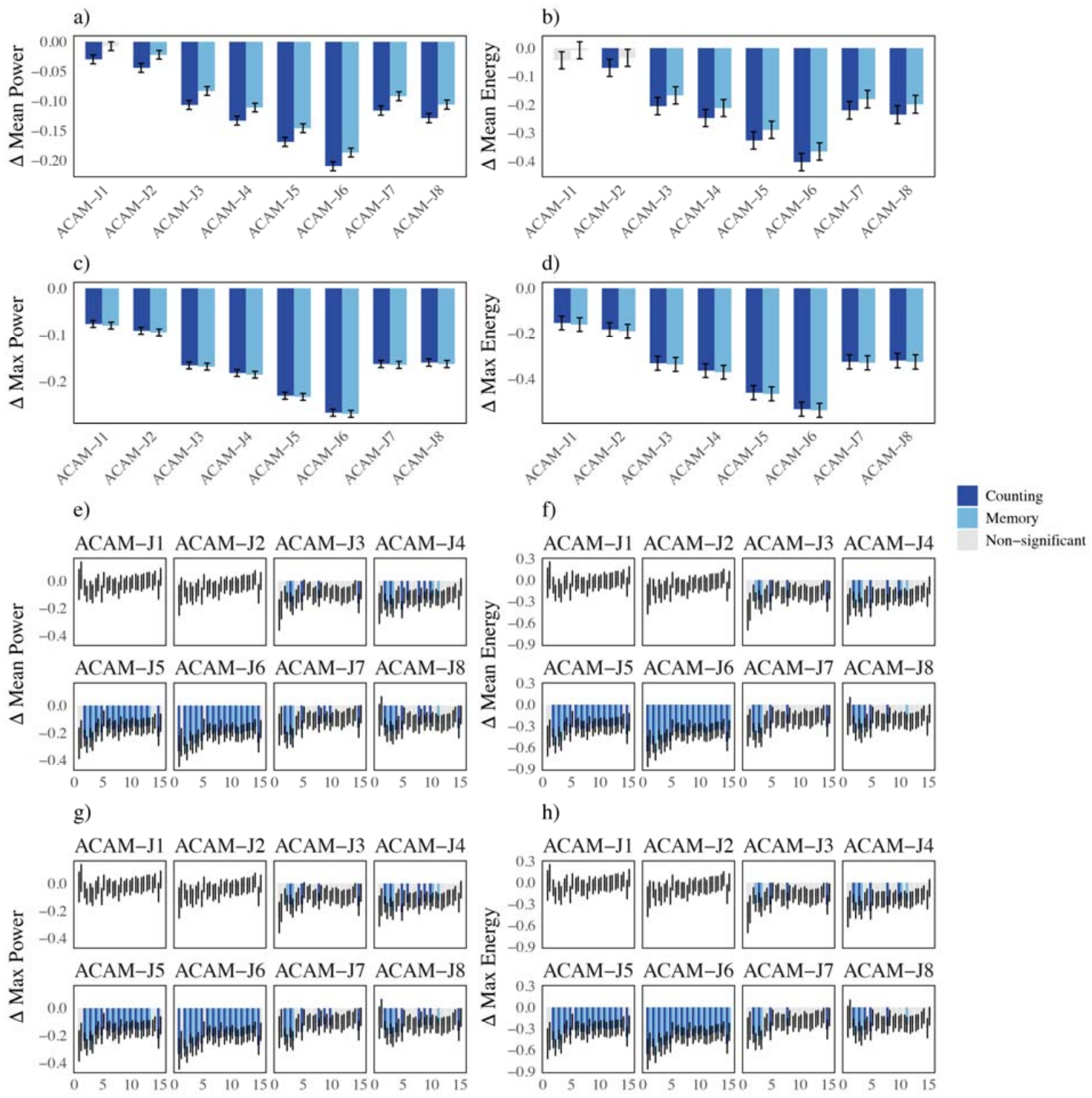

Figure S8. Global cortical dynamics compared to counting and memory control conditions (N=20). (a-d) Compared to all control conditions, most ACAM-J (except for ACAM-J1 and ACAM-J2) showed reduced (a-b) mean and (c-d) max power/energy. (e-h) There were subtle differences between ACAM-J, with minimal differences for ACAM-J1 and ACAM-J2. (e-f) Mean power/energy showed the most notable differences in eigengroup activations during ACAM-J5-6. In contrast, (g-h) max power/energy revealed differences in nearly all eigengroups between ACAM-J3 and ACAM-J8 when compared to both control conditions.

bioRxiv preprint doi: https://doi.org/10.1101/2025.11.12.688050; this version posted November 13, 2025. The copyright holder for this preprint (which was not certified by peer review) is the author/funder, who has granted bioRxiv a license to display the preprint in perpetuity. It is made available under aCC-BY 4.0 International license.

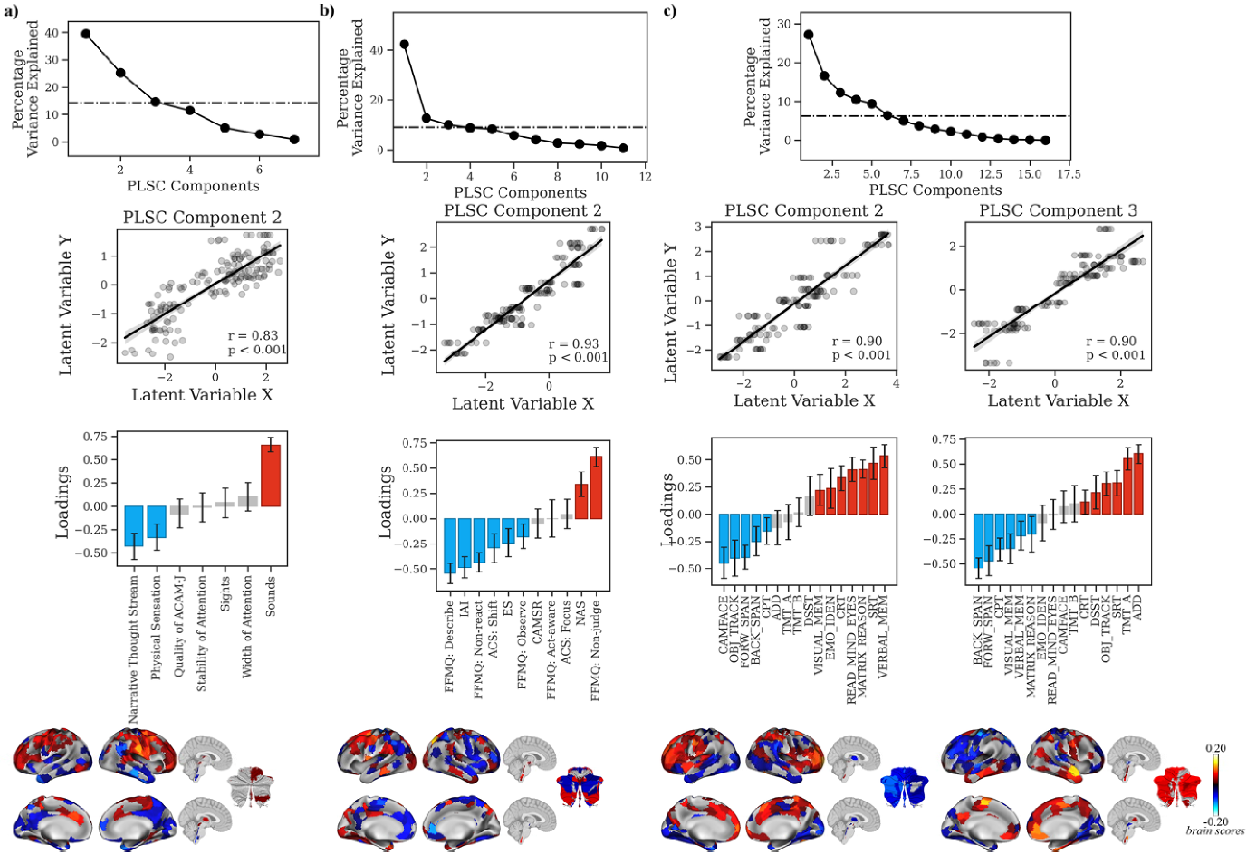

Figure S10. Additional multivariate relationship between ReHo during ACAM-J and behavioral patterns in the full sample (N=20). LV2 captured (a) ACAM-J phenomenology, with dorsal attention regions covaried with stability/width of attention, quality of ACAM-J, narrative thought stream, and physical sensations, and ventral sensory regions covaried with sounds; (b) meditative traits, with visuo-temporal, dorsolateral prefrontal cortex, and brainstem covaried with non-attachment/non-judgment and focus, and medial/lateral prefrontal regions aligned with general mindfulness; and (c) cognition, with PFC covaried with general cognition and visuo-temporal/basal ganglia covaried with working memory for latent variable 2 (LV2) and visual, prefrontal regions, brainstem, and cerebellum, covary with social function while lateral temporal and somatomotor regions covary with working memory functions. Error bars represent standard errors.

bioRxiv preprint doi: https://doi.org/10.1101/2025.11.12.688050; this version posted November 13, 2025. The copyright holder for this preprint (which was not certified by peer review) is the author/funder, who has granted bioRxiv a license to display the preprint in perpetuity. It is made available under aCC-BY 4.0 International license.

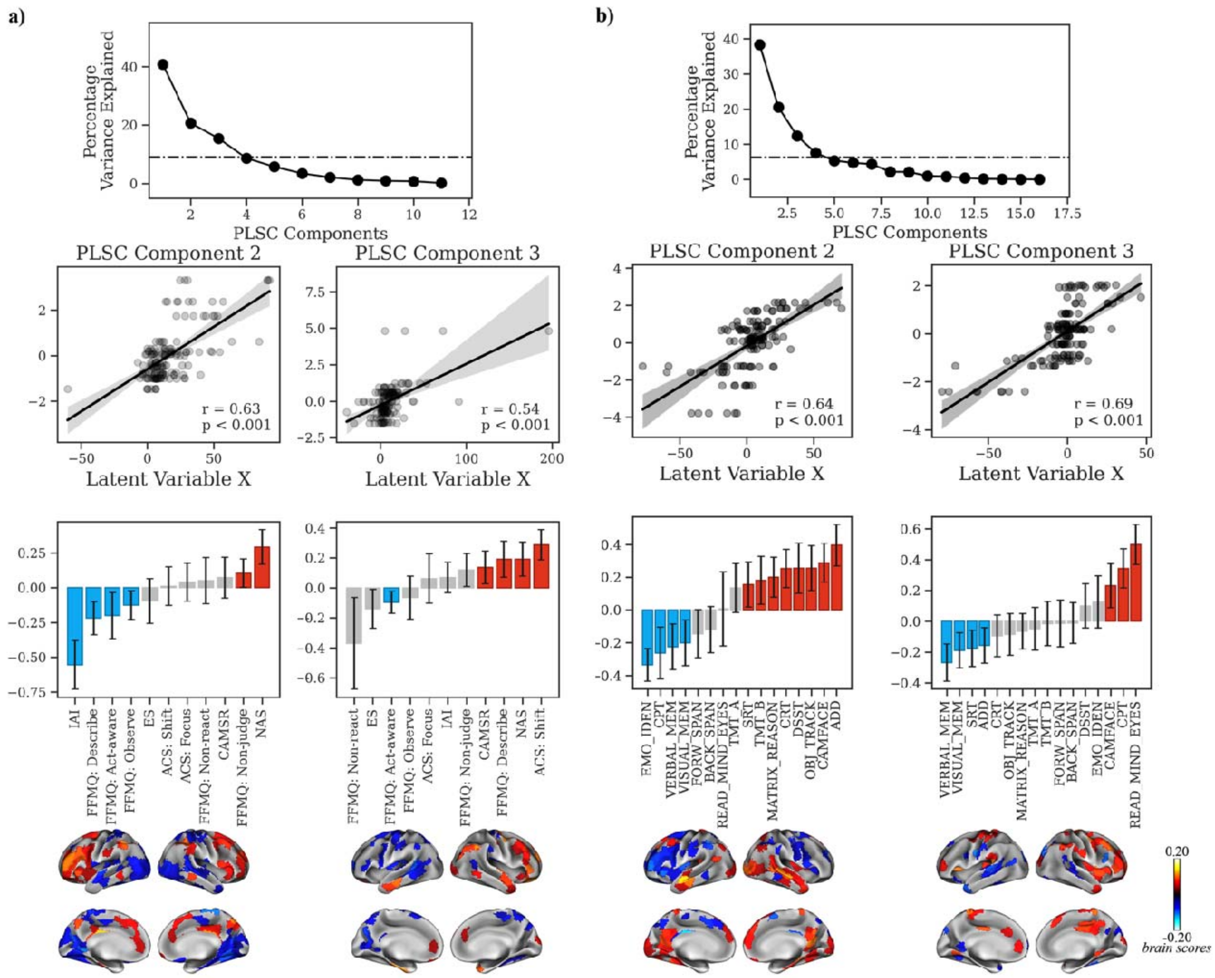

Figure S11. Multivariate relationship between G1 during ACAM-J and behavioral patterns. During ACAM-J, (a) For LV2 (left), visuo-temporal regions coavaried with nonattachment and dorsolateral PFC covaried with mindfulness. For LV 3 (right), visual, somatomotor, and posterior reigons covaried with awareness while frontotemporal regions covaried with mindfulness abilities (b) For cognition, LV2 showed covariance between general cognitive functions and visuo-temporal regions while working memory covaried with dorsolateral PFC. For LV 3, social function covaried with medial regions and PFC while working memory and sustained attention covaried with visual regions.

bioRxiv preprint doi: https://doi.org/10.1101/2025.11.12.688050; this version posted November 13, 2025. The copyright holder for this preprint (which was not certified by peer review) is the author/funder, who has granted bioRxiv a license to display the preprint in perpetuity. It is made available under aCC-BY 4.0 International license.

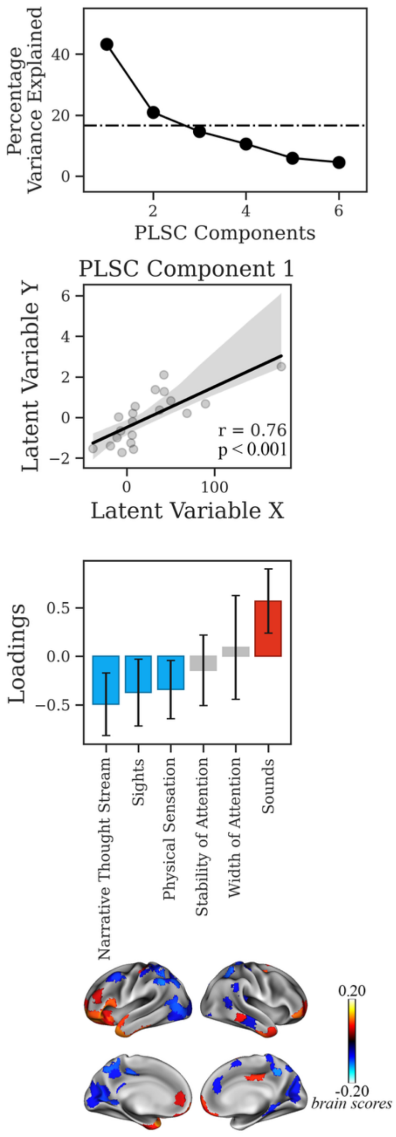

Figure S12. Multivariate relationship between G1 during control conditions and behavioral patterns. For the memory task, LV1 showed significant covariance between phenomenology and G1 values. Sensation of rounds covaried with G1 values in the PFC and temporal poles while other sensations (sights, physical sensations, narrative thought stream) covaried with G1 values in the visual and posterior regions.

bioRxiv preprint doi: https://doi.org/10.1101/2025.11.12.688050; this version posted November 13, 2025. The copyright holder for this preprint (which was not certified by peer review) is the author/funder, who has granted bioRxiv a license to display the preprint in perpetuity. It is made available under aCC-BY 4.0 International license.

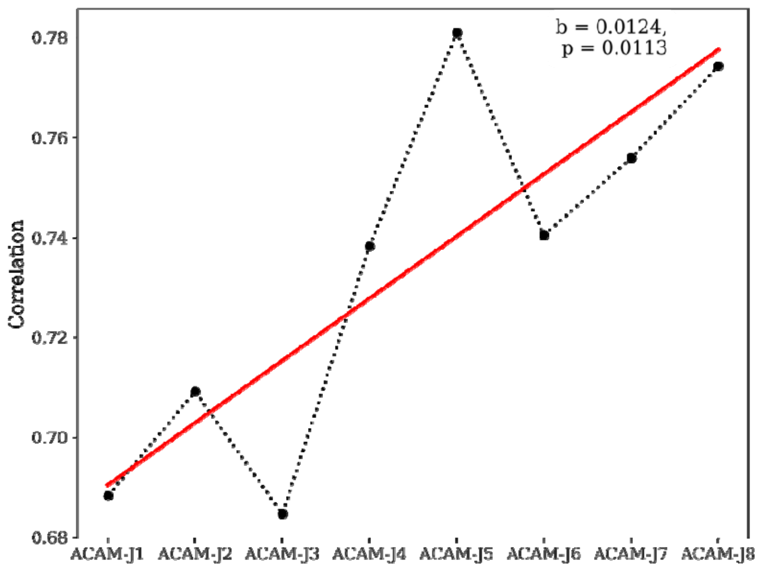

Figure S13. Spatial similarity between delta-ReHo maps of Sutta-style and TWIM-style ACAM-J across ACAM-J. Each point represents the spatial correlation at a ACAM-J. The red line indicates the best-fit linear regression (slope = 0.0124, = 0.0113), suggesting a significant positive trend in neural pattern similarity as ACAM-J deepens. This supports the hypothesis that TWIM-style and Sutta-style ACAM-J converge on similar neural signatures over the progression of meditative absorption.

bioRxiv preprint doi: https://doi.org/10.1101/2025.11.12.688050; this version posted November 13, 2025. The copyright holder for this preprint (which was not certified by peer review) is the author/funder, who has granted bioRxiv a license to display the preprint in perpetuity. It is made available under aCC-BY 4.0 International license.

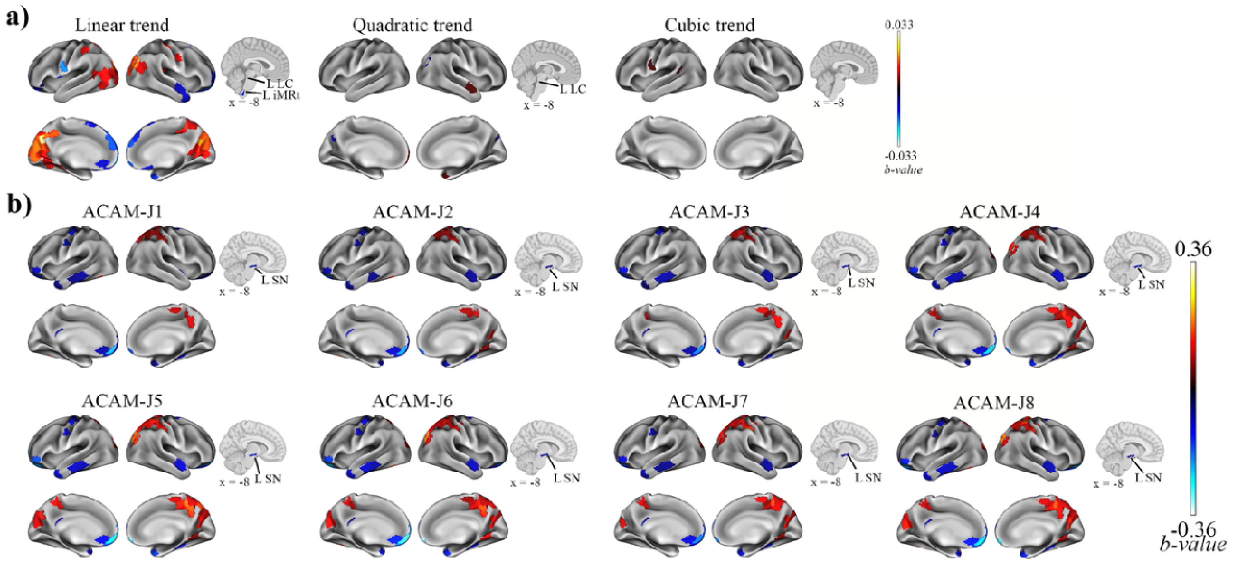

Figure S14. Distinct ReHo patterns intermediate ACAM-J. Excluding two deep ACAM-J practitioners did not influence the ReHo patterns in the results. (a) Polynomial trend analysis showing brain regions exhibiting significant linear, quadratic, and cubic changes in ReHo across ACAM-J. A significant linear trend is observed in medial prefrontal and posterior visual regions, suggesting progressive changes in brain activity across ACAM-J. Although quadratic or cubic trends were detected in very few parcels, their influence is not as remarkable as those of the linear trends. (b) Each ACAM-J showed consistent spatial patterns, particularly in the lateral temporal, prefrontal, and parietal cortices. Blue indicates decreased ReHo, while red indicates increased ReHo relative to the control condition. These findings confirm that excluding deep-level ACAM-J practitioners did not qualitatively alter the observed ReHo trajectories, supporting the robustness of the main results within the intermediate-level cohort.

bioRxiv preprint doi: https://doi.org/10.1101/2025.11.12.688050; this version posted November 13, 2025. The copyright holder for this preprint (which was not certified by peer review) is the author/funder, who has granted bioRxiv a license to display the preprint in perpetuity. It is made available under aCC-BY 4.0 International license.

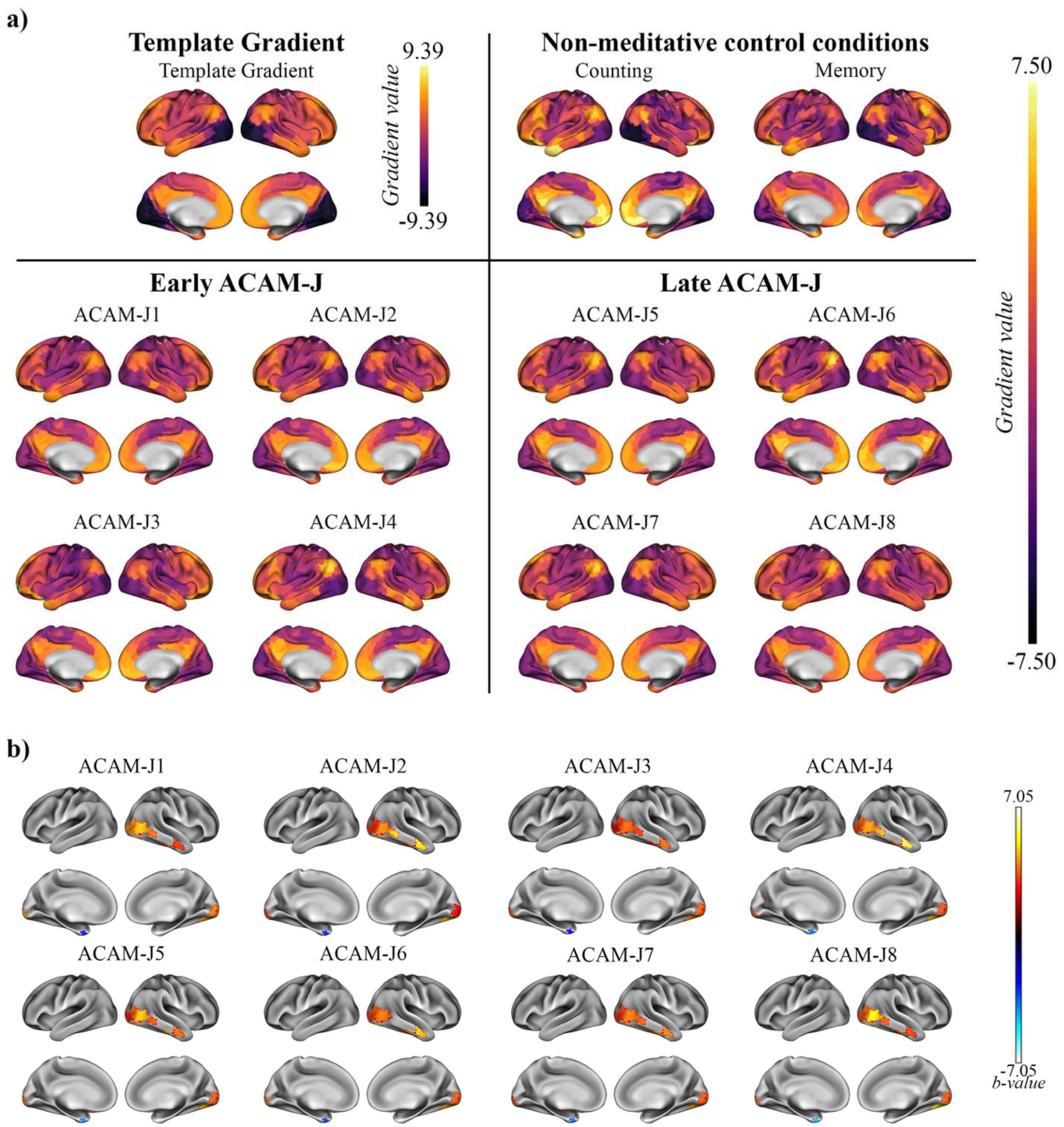

Figure S15. G1 dynamics across meditative and control conditions in intermediate ACAM-J practitioners (N = 18). (a) Template principal gradient (G1) used for gradient alignment in this study, and average gradient maps for the non-meditative control conditions (counting, memory) and ACAM-J, grouped into early and late absorption phases. (b) Contrasts between individual ACAM-J and the compositive control condition reveal robust changes along G1 in visual and temporal hubs highlighting regional-specific reorganization of large-scale cortical hierarchy during ACAM-J.

bioRxiv preprint doi: https://doi.org/10.1101/2025.11.12.688050; this version posted November 13, 2025. The copyright holder for this preprint (which was not certified by peer review) is the author/funder, who has granted bioRxiv a license to display the preprint in perpetuity. It is made available under aCC-BY 4.0 International license.

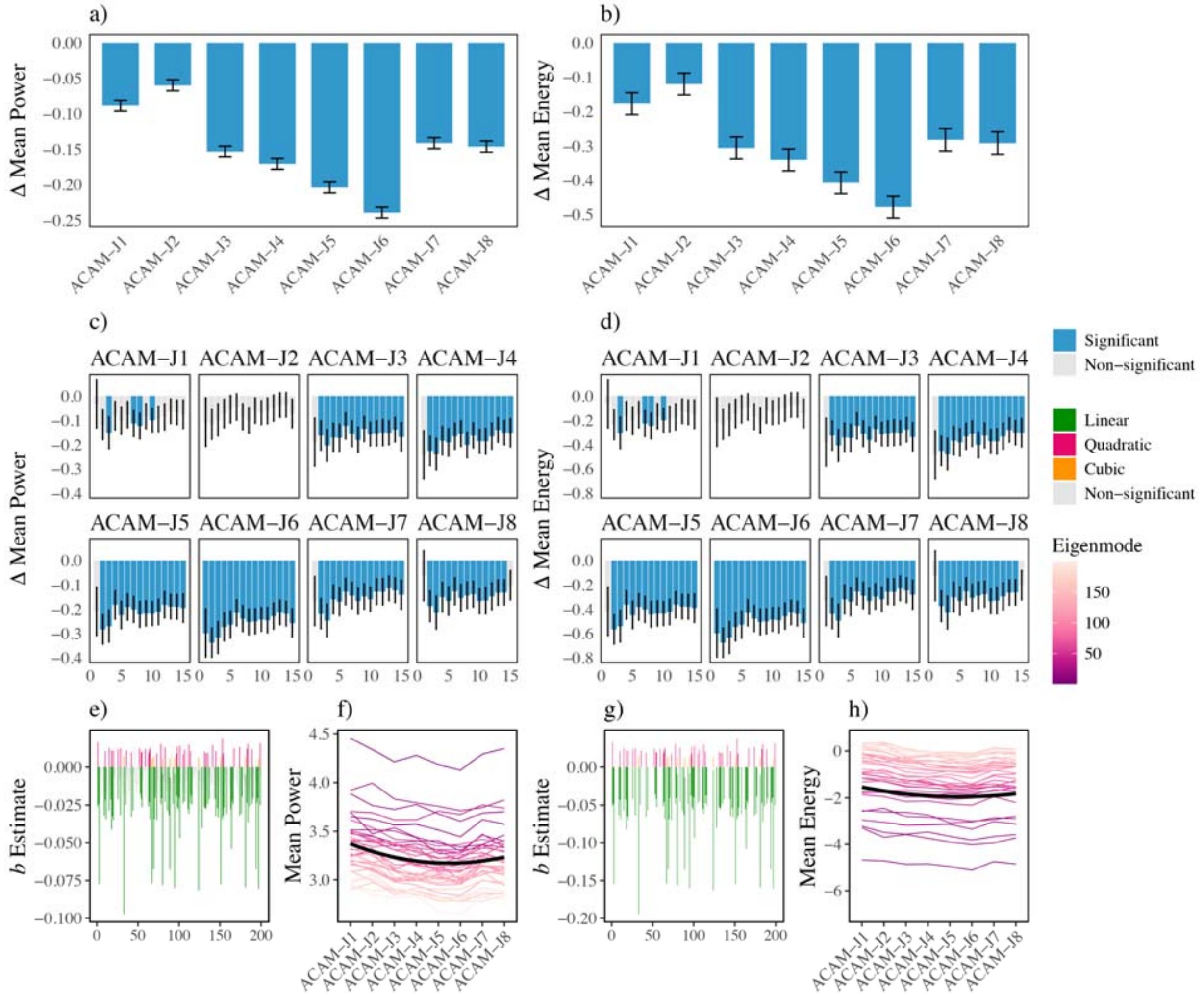

Figure S16. Geometric eigenmode decomposition during intermediate ACAM-J (N=18). (a–b) Overall mean/max power and energy reduced during ACAM-J compared with controls, except for ACAM-J1. (c-d) Most eigengroup differences were found in ACAM-J5-6. (e-h) Polynomial trends revealed consistent negative linear and positive quadratic trajectories across ACAM-J.

bioRxiv preprint doi: https://doi.org/10.1101/2025.11.12.688050; this version posted November 13, 2025. The copyright holder for this preprint (which was not certified by peer review) is the author/funder, who has granted bioRxiv a license to display the preprint in perpetuity. It is made available under aCC-BY 4.0 International license.

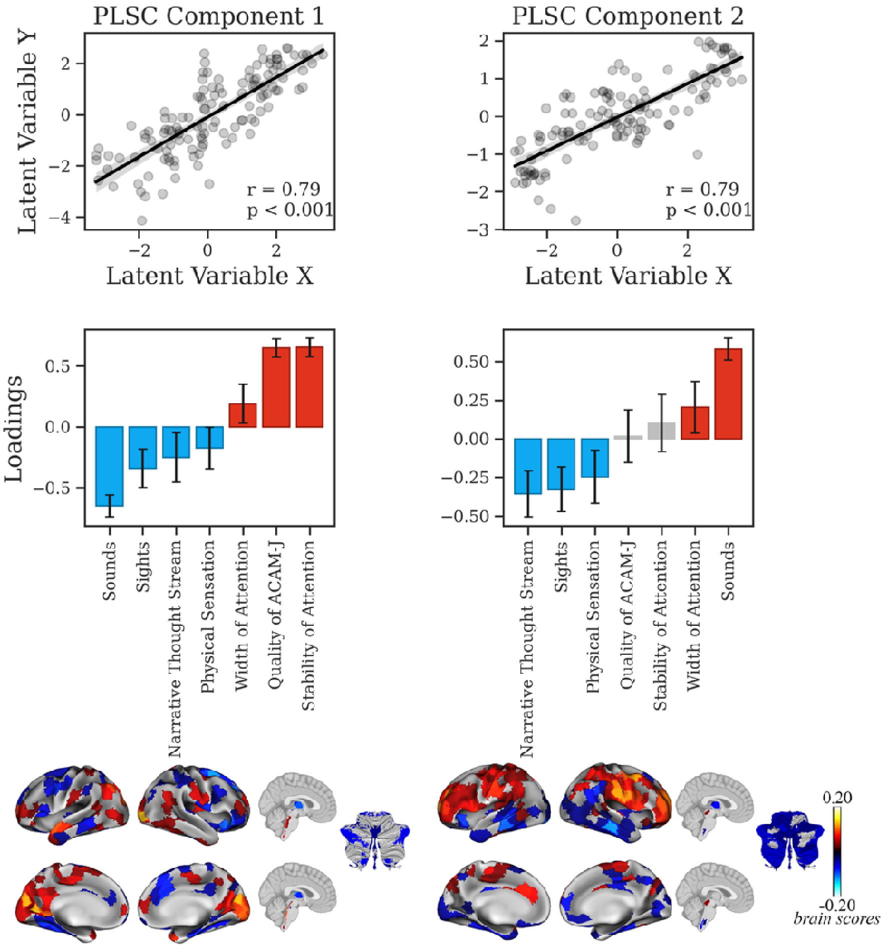

Figure S17. Multivariate relationship between ReHo and phenomenology during intermediate ACAM-J (N=18). PLSC identified two LVs linking ReHo with phenomenology. LV1 (left) primarily dissociated sensory and narrative processes (negative loadings: sounds, sights, narrative thought stream, physical sensation) from attentional qualities (positive loadings: width, stability of attention, quality of ACAM-J). In contrast, LV2 highlighted sensory salience, with sounds showing the strongest positive loading. Brain scores maps for LVs 1 and 2 reveal distributed cortical and subcortical systems covarying with phenomenological dimensions. Warm and cool colors represent positive and negative brain scores, respectively.

bioRxiv preprint doi: https://doi.org/10.1101/2025.11.12.688050; this version posted November 13, 2025. The copyright holder for this preprint (which was not certified by peer review) is the author/funder, who has granted bioRxiv a license to display the preprint in perpetuity. It is made available under aCC-BY 4.0 International license.

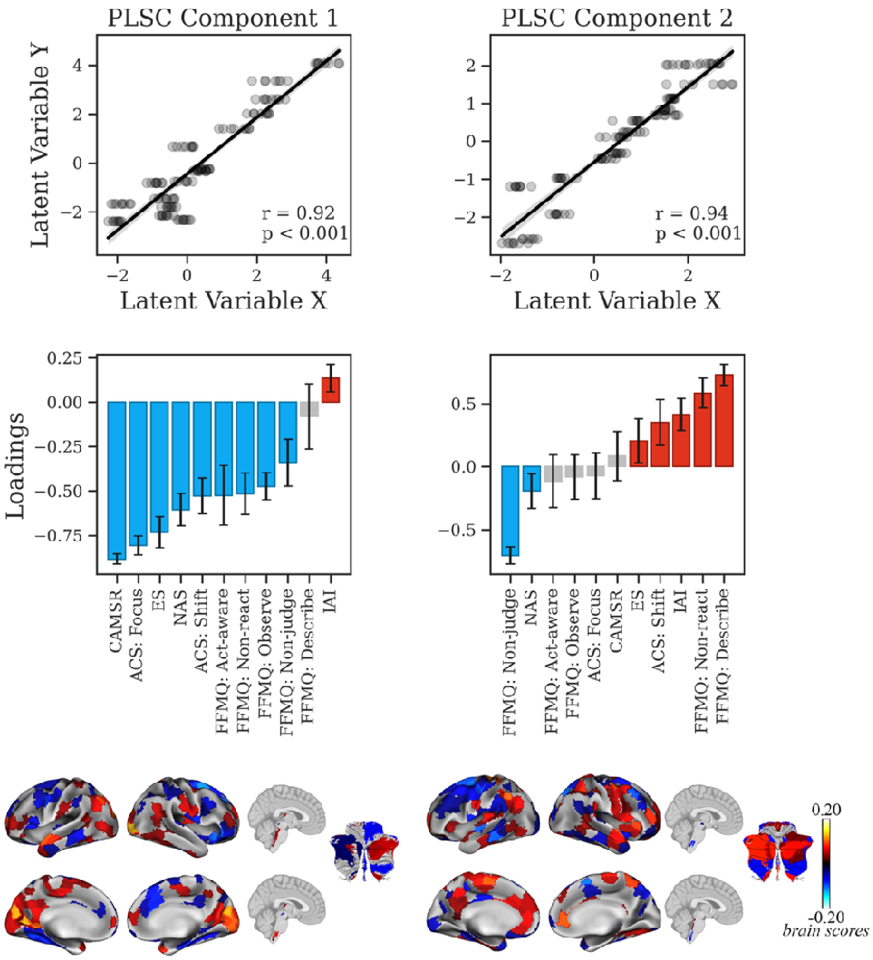

Figure S18. Multivariate relationship between ReHo and meditative traits during intermediate ACAM-J (N=18). PLSC identified two LVs linking ReHo with meditative traits. LV1 (left) primarily dissociated interoceptions (negative loadings: interoceptive awareness) from all other general mindfulness abilities (positrive loadings). In contrast, LV2 highlighted non-attachement (negative loadings) from mindfulness with equanimity (positive loadings). Brain scores maps for LVs 1 and 2 reveal distributed cortical and subcortical systems covarying with meditative traits. Warm and cool colors represent positive and negative brain scores, respectively.

bioRxiv preprint doi: https://doi.org/10.1101/2025.11.12.688050; this version posted November 13, 2025. The copyright holder for this preprint (which was not certified by peer review) is the author/funder, who has granted bioRxiv a license to display the preprint in perpetuity. It is made available under aCC-BY 4.0 International license.

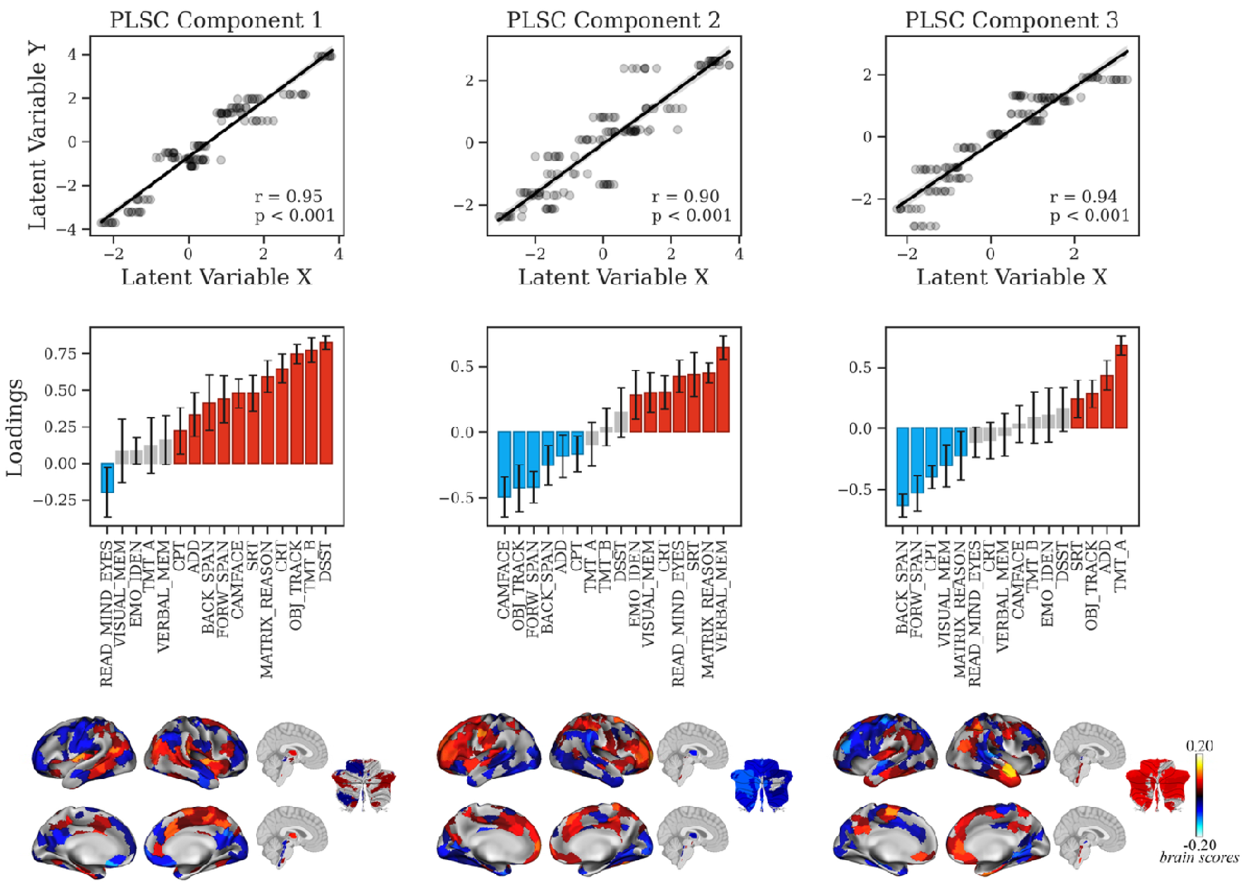

Figure S19. Multivariate relationship between ReHo and cognitive performance during intermediate ACAM-J (N=18). PLSC identified three LVs linking ReHo with cognitive performance. LV1 reflected a general cognitive proficiency axis, with strong positive loadings for most domains and negative loadings for social cognition (reading the mind in the eyes). LV2 dissociated socio-affective processing (negative loadings) from executive and attentional abilities (positive loadings). LV3 emphasized working memory and attentional control (positive loadings) relative to verbal memory and mentalizing abilities (negative loadings). Brain score maps for LVs 1 to 3 revealed distributed cortical and subcortical networks covarying with these distinct cognitive dimensions, with warm and cool colors indicating positive and negative brain scores, respectively.

bioRxiv preprint doi: https://doi.org/10.1101/2025.11.12.688050; this version posted November 13, 2025. The copyright holder for this preprint (which was not certified by peer review) is the author/funder, who has granted bioRxiv a license to display the preprint in perpetuity. It is made available under aCC-BY 4.0 International license.

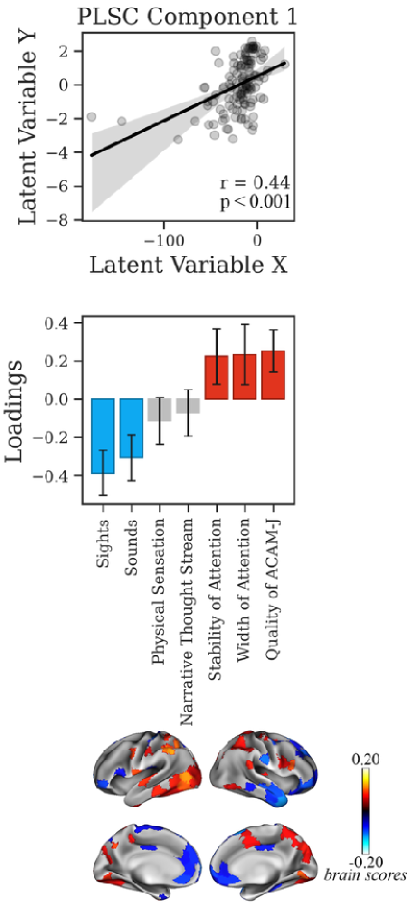

Figure S20. Multivariate relationship between G1 and phenomenology during intermediate ACAM-J (N=18). PLSC identified one LV linking G1 with phenomenology. Negative loadings were observed for attentional qualities (quality of ACAM-J, stability, width of attention), whereas positive loadings reflected sensory processes (sounds, sights,). Brain score maps revealed distributed cortical systems covarying with this phenomenological dissociation, with warm and cool colors representing positive and negative brain scores, respectively.

bioRxiv preprint doi: https://doi.org/10.1101/2025.11.12.688050; this version posted November 13, 2025. The copyright holder for this preprint (which was not certified by peer review) is the author/funder, who has granted bioRxiv a license to display the preprint in perpetuity. It is made available under aCC-BY 4.0 International license.

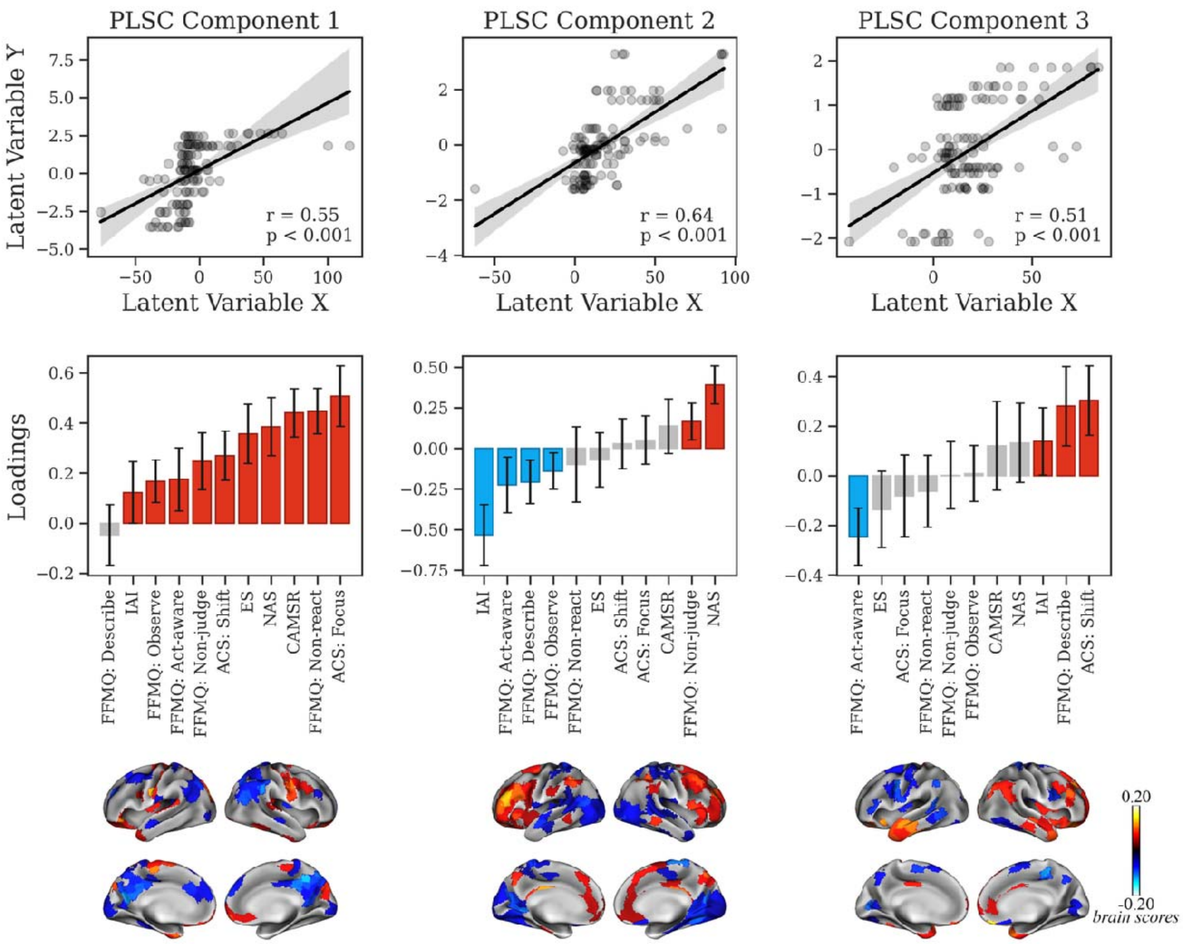

Figure S21. Multivariate relationship between G1 and meditative traits during intermediate ACAM-J (N=18). PLSC revealed three significant LVs linking large-scale cortical gradient organization with meditative traits. LV1 primarily reflected a broad positive association. LV2 dissociated mindfulness (negative loadings) from non-reactivity and non-attachment (positive loadings). LV3 further emphasized equanimity relative to descriptive mindfulness and attentional shifting (positive loadings). Brain score maps for LVs 1 to 3 revealed distributed cortical networks covarying with these trait dimensions, with warm and cool colors indicating positive and negative brain scores, respectively.

bioRxiv preprint doi: https://doi.org/10.1101/2025.11.12.688050; this version posted November 13, 2025. The copyright holder for this preprint (which was not certified by peer review) is the author/funder, who has granted bioRxiv a license to display the preprint in perpetuity. It is made available under aCC-BY 4.0 International license.

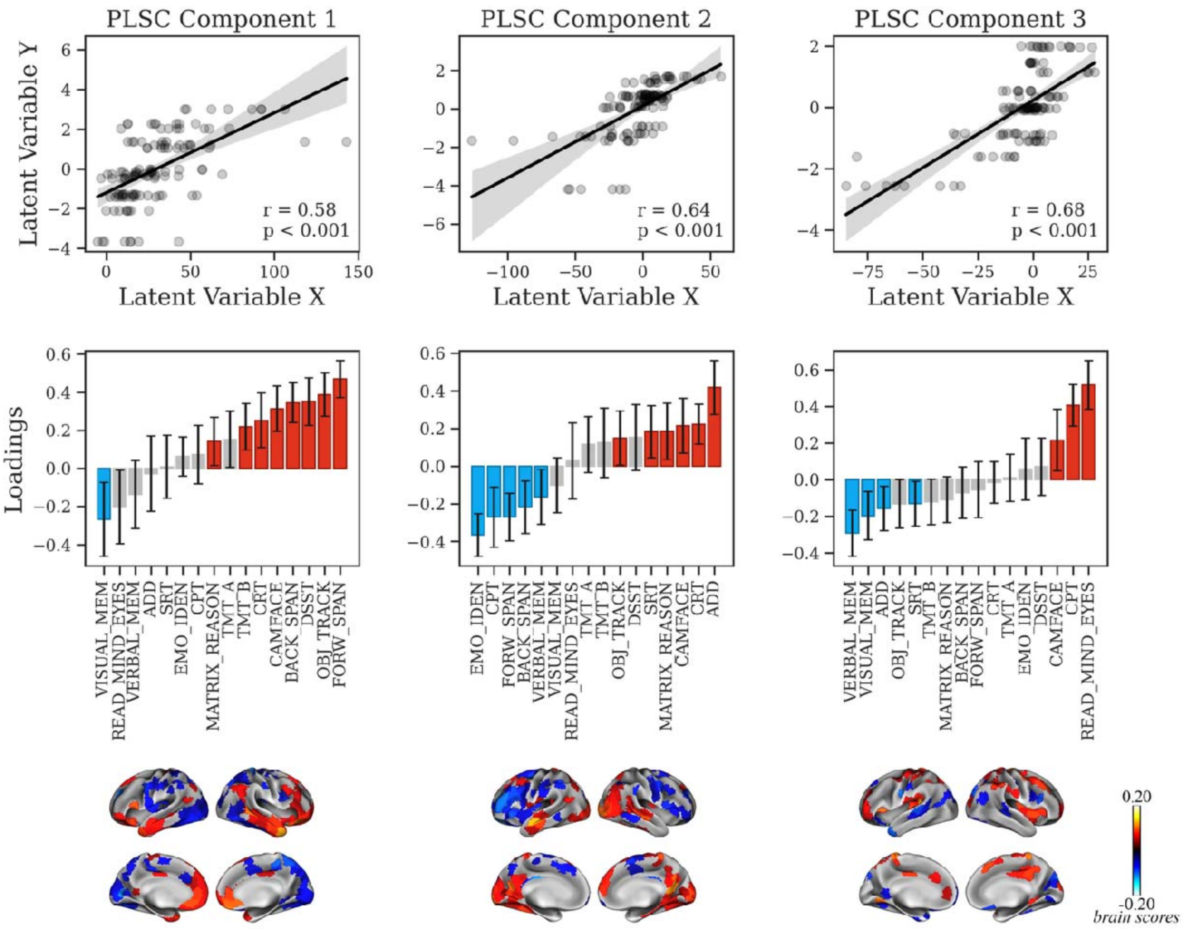

Figure S22. Multivariate relationship between G1 and cognition during intermediate ACAM-J (N=18). PLSC identified three significant LVs linking G1 with cognitive performance. LV1 reflected general cognitive perfofrmance, with strongest positive loadings for working memory, executive function, and attentional control, contrasted against contributions from verbal and visual memory (negative loadings). LV2 dissociated socio-affective processes (negative loadings) from executive reasoning and attentional measures (positive loadings). LV3 emphasized socio-cognitive and attentional skills (positive loadings) relative to verbal memory (negative loadings). Brain score maps for LVs 1 to 3 revealed distributed cortical and subcortical systems covarying with these cognitive dimensions, with warm and cool colors denoting positive and negative brain scores, respectively.

bioRxiv preprint doi: https://doi.org/10.1101/2025.11.12.688050; this version posted November 13, 2025. The copyright holder for this preprint (which was not certified by peer review) is the author/funder, who has granted bioRxiv a license to display the preprint in perpetuity. It is made available under aCC-BY 4.0 International license.

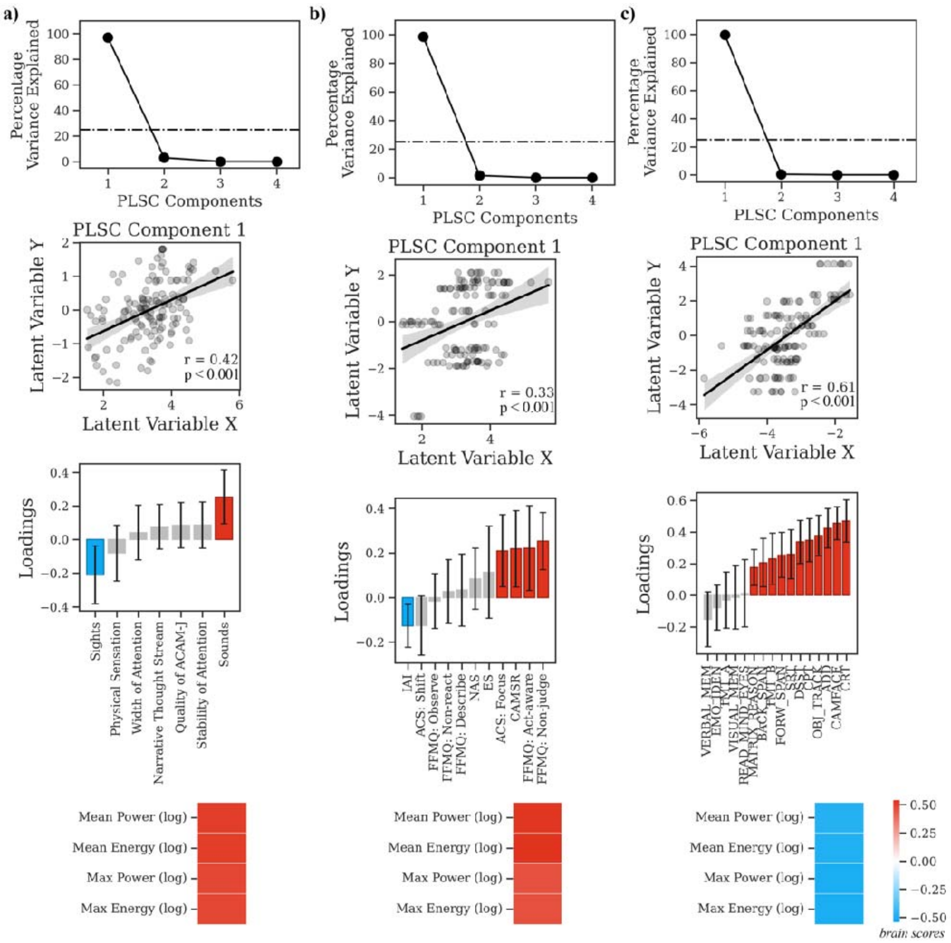

Figure S23. Multivariate relationship between global eigenmode activations and behavioral patterns during intermediate ACAM-J (N=18). PLSC revealed one significant LV in each domain linking eigenmode organization with (a) phenomenology, (b) meditative traits, and (c) cognitive performance. For phenomenology (a), the LV dissociated sensation of sights (negative loadings) from sounds (positive loadings). For meditative traits (b), the LV highlighted broad positive associations with trait mindfulness (positive loadings), contrasted against interoceptive awareness (negative loadings). For cognition (c), the LV captured a general cognitive performance axis, with positive loadings spanning working memory, executive function, and attentional control. Brain score maps indicated distributed cortical systems covarying with each domain, while bar plots show consistent positive relationships between eigenmode metrics (mean/max power and energy) and latent brain–behavior associations.

bioRxiv preprint doi: https://doi.org/10.1101/2025.11.12.688050; this version posted November 13, 2025. The copyright holder for this preprint (which was not certified by peer review) is the author/funder, who has granted bioRxiv a license to display the preprint in perpetuity. It is made available under aCC-BY 4.0 International license.

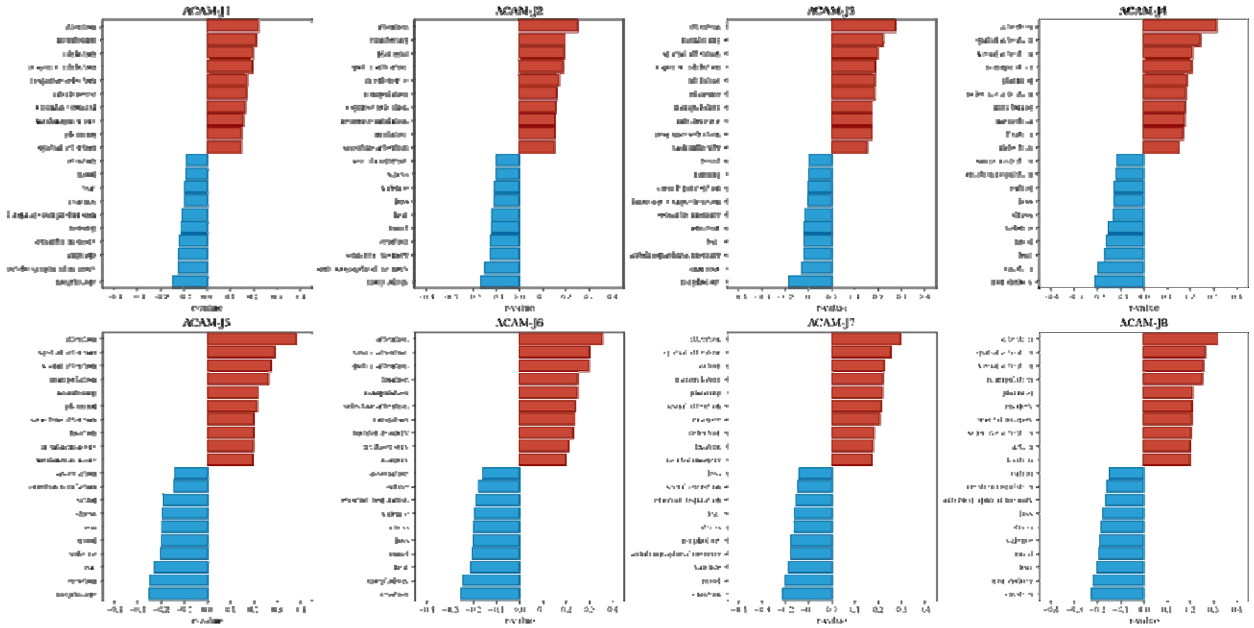

Figure S24. Reorganization of cognitive and affective domains during intermediate ACAM-J (N=18). For each ACAM-J, bar plots display the top ten functions with positive (red) and negative (blue) associations derived from Neurosynth decoding. Positive associations were dominated by attentional, executive, visuospatial, and action-related domains (e.g., sustained and selective attention, planning, imagery). In contrast, negative associations consistently involved affective, autobiographical, and bodily–social domains (e.g., emotion, mood, stress, fear, autobiographical memory, social cognition, eating, morphology).

bioRxiv preprint doi: https://doi.org/10.1101/2025.11.12.688050; this version posted November 13, 2025. The copyright holder for this preprint (which was not certified by peer review) is the author/funder, who has granted bioRxiv a license to display the preprint in perpetuity. It is made available under aCC-BY 4.0 International license.

Table S1. Average time spent in each ACAM-J after data cleaning.

**ACAM-J**	**Duration in minutes (M ± SD)**
ACAM-J1	4.04 ± 4.29
ACAM-J2	3.63 ± 1.38
ACAM-J3	3.38 ± 1.04
ACAM-J4	3.38 ± 1.24
ACAM-J5	3.25 ± 1.47
ACAM-J6	4.08 ± 4.69
ACAM-J7	3.21 ± 1.38
ACAM-J8	3.25 ± 1.63

bioRxiv preprint doi: https://doi.org/10.1101/2025.11.12.688050; this version posted November 13, 2025. The copyright holder for this preprint (which was not certified by peer review) is the author/funder, who has granted bioRxiv a license to display the preprint in perpetuity. It is made available under aCC-BY 4.0 International license.

Table S2. Description of cognitive tasks

**Task Name**	**Abbreviation**	**Description**
Simple Reaction Time	SRT	Participant presses a key whenever a green square appears. Measures basic psychomotor response speed.
Choice Reaction Task	CRT	Participant indicates the direction of the arrow that is a different color from the rest. Measures processing speed, response selection/inhibition, and attention.
Digit Symbol Matching	DSST	Participant matches as many number/symbol pairs as possible in 90 seconds. Measures processing speed.
Trail Making Test Part A	TMT_A	Participant connects a series of circles with numbers in ascending order. Measures processing speed.
Trail Making Test Part B	TMT_B	Participant connects a series of circles with numbers and letters in alternating numerical and alphabetical order. Measures processing speed and task-switching (or cognitive flexibility).
Continuous Performance Test	CPT	Participant presses a button for each city image and withholds button presses for mountain images. Measures cognitive control, sustained attention, and response inhibition.
Paced Serial Addition Test	ADD	Participant adds sequentially presented pairs of numbers and judges whether their sum is less than or greater than 10. Measures sustained attention and working memory.
Forward Digit Span	FORW_SPAN	Participant is shown a series of numbers in increasing length and asked to recall the numbers in the order displayed.
Backward Digit Span	BACK_SPAN	asked participant is shown a series of numbers in increasing length and asked to recall the numbers in reverse order.
Multiple Object Tracking	OBJ_TRACK	Participant tracks a set of hidden dots across the screen. The dots increase in number and speed as the test progresses. Measures visuospatial attention and visual working memory.
Matrix Reasoning	MATRIX_REASON	Participant selects which smaller image completes the pattern in the larger image. Measures visual reasoning and general cognitive ability.
Verbal Paired Associates Memory	VERBAL_MEM	Participant memorizes a set of word pairs. Then, after completing TMB Digit Symbol Matching (90sec), the participant selects which words were paired together.
Visual Paired Associates Memory	VISUAL_MEM	Participant memorizes a set of picture pairs. Then, after completing TMB Digit Symbol Matching (90sec), the participant selects which pictures were paired together.
Cambridge Face Memory Test	CAMFACE	Participant memorizes a set of six faces. Then, the participant identifies the faces learned in the memorization phase from images where lighting and face angle vary. Measures unfamiliar face recognition.
Multiracial Emotion Identification	EMO_IDEN	Participant picks which of four basic emotions (happy, fearful, sad, or angry) best describes a face. Measures face emotion recognition.
Reading the Mind in the Eyes	READ_MIND_EYES	Participant judges which of four complex emotion words best describes a pair of eyes. Measures mental state inferencing and theory of mind.

bioRxiv preprint doi: https://doi.org/10.1101/2025.11.12.688050; this version posted November 13, 2025. The copyright holder for this preprint (which was not certified by peer review) is the author/funder, who has granted bioRxiv a license to display the preprint in perpetuity. It is made available under aCC-BY 4.0 International license.

Table S3. Significant mean/max eigenmode power/energy during ACAM-J compared to non-meditative control conditions in the full sample

Control Condition	ACAM-J	Mean Power	Mean Energy	Max Power	Max Energy
Composite	ACAM-J1	-	-	3, 8, 10	3, 8, 10
	ACAM-J2	-	-	2, 3, 8, 10	2, 3, 8, 10
	ACAM-J3	2, 3	2, 3	10, 11, 12, 13, 14, 15, 2, 3, 4, 5, 6, 7, 8, 9	10, 11, 12, 13, 14, 15, 2, 3, 4, 5, 6, 7, 8, 9
	ACAM-J4	2, 3, 5, 6, 8, 9, 10	2, 3, 5, 8, 10	1, 2, 3, 4, 5, 6, 7, 8, 9, 10, 11, 12, 13, 14, 15	1, 2, 3, 4, 5, 6, 7, 8, 9, 10, 11, 12, 13, 14, 15
	ACAM-J5	2, 3, 4, 5, 6, 7, 8, 9, 10, 11, 12, 13, 15	2, 3, 4, 5, 6, 7, 8, 9, 10, 11, 12, 13, 15,	1, 2, 3, 4, 5, 6, 7, 8, 9, 10, 11, 12, 13, 14, 15	1, 2, 3, 4, 5, 6, 7, 8, 9, 10, 11, 12, 13, 14, 15
	ACAM-J6	1, 2, 3, 4, 5, 6, 7, 8, 9, 10, 11, 12, 13, 14, 15	1, 2, 3, 4, 5, 6, 7, 8, 9, 10, 11, 12, 13, 14, 15	1, 2, 3, 4, 5, 6, 7, 8, 9, 10, 11, 12, 13, 14, 15	1, 2, 3, 4, 5, 6, 7, 8, 9, 10, 11, 12, 13, 14, 15
	ACAM-J7	2, 3, 10	2, 3	2, 3, 4, 5, 6, 7, 8, 9, 10, 11, 12, 13, 14, 15	2, 3, 4, 5, 6, 7, 8, 9, 10, 11, 12, 13, 14, 15
	ACAM-J8	2, 3, 4, 10	2, 3	2, 3, 4, 5, 6, 7, 8, 9, 10, 11, 12, 13, 14	2, 3, 4, 5, 6, 7, 8, 9, 10, 11, 12, 13, 14
Counting	ACAM-J1	-	-	8	8
	ACAM-J2	-	-	8	8
	ACAM-J3	3, 5, 8, 15	3, 5, 8, 15	2, 3, 4, 5, 6, 7, 8, 9, 10, 11, 12, 13, 14, 15	2, 3, 4, 5, 6, 7, 8, 9, 10, 11, 12, 13, 14, 15
	ACAM-J4	2, 3, 5, 6, 8, 9, 10	2, 3, 5, 8, 9, 10	1, 2, 3, 4, 5, 6, 7, 8, 9, 10, 11, 12, 13, 14, 15	1, 2, 3, 4, 5, 6, 7, 8, 9, 10, 11, 12, 13, 14, 15
	ACAM-J5	2, 3, 4, 5, 6, 7, 8, 9, 10, 11, 12, 13, 15	2, 3, 4, 5, 6, 7, 8, 9, 10, 11, 12, 13, 14, 15	1, 2, 3, 4, 5, 6, 7, 8, 9, 10, 11, 12, 13, 14, 15	1, 2, 3, 4, 5, 6, 7, 8, 9, 10, 11, 12, 13, 14, 15
	ACAM-J6	1, 2, 3, 4, 5, 6, 7, 8, 9, 10, 11, 12, 13, 14, 15	1, 2, 3, 4, 5, 6, 7, 8, 9, 10, 11, 12, 13, 14, 15	1, 2, 3, 4, 5, 6, 7, 8, 9, 10, 11, 12, 13, 14, 15	1, 2, 3, 4, 5, 6, 7, 8, 9, 10, 11, 12, 13, 14, 15
	ACAM-J7	2, 3, 5, 8, 9, 10, 15	2, 3, 5, 8, 15	2, 3, 4, 5, 6, 7, 8, 9, 10, 11, 12, 13, 14, 15	2, 3, 4, 5, 6, 7, 8, 9, 10, 11, 12, 13, 14, 15
	ACAM-J8	2, 3, 4, 5, 8, 9, 10, 15	2, 3, 4, 5, 8	2, 3, 4, 5, 6, 7, 8, 9, 10, 11, 12, 13, 14, 15	2, 3, 4, 5, 6, 7, 8, 9, 10, 11, 12, 13, 14, 15
Memory	ACAM-J1	-	-	2, 3, 7, 10	2, 3, 7, 10
	ACAM-J2	-	-	2, 3, 7, 10	2, 3, 7, 10
	ACAM-J3	2, 3	2, 3	2, 3, 4, 5, 6, 7, 8, 9, 10, 11, 12, 13, 14, 15	2, 3, 4, 5, 6, 7, 8, 9, 10, 11, 12, 13, 14, 15
	ACAM-J4	2, 3, 10, 11,	2, 3, 10, 11,	1, 2, 3, 4, 5, 6, 7, 8, 9, 10, 11, 12, 13, 14	1, 2, 3, 4, 5, 6, 7, 8, 9, 10, 11, 12, 13, 14
	ACAM-J5	2, 3, 4, 6, 7, 8, 9, 10, 11, 12, 13	2, 3, 4, 6, 7, 8, 9, 10, 11, 12, 13	1, 2, 3, 4, 5, 6, 7, 8, 9, 10, 11, 12, 13, 14, 15	1, 2, 3, 4, 5, 6, 7, 8, 9, 10, 11, 12, 13, 14, 15
	ACAM-J6	2, 3, 4, 5, 6, 7, 8, 9, 10, 11, 12, 13, 14	1, 2, 3, 4, 5, 6, 7, 8, 9, 10, 11, 12, 13, 14	1, 2, 3, 4, 5, 6, 7, 8, 9, 10, 11, 12, 13, 14, 15	1, 2, 3, 4, 5, 6, 7, 8, 9, 10, 11, 12, 13, 14, 15
	ACAM-J7	2, 3	2, 3	2, 3, 4, 5, 6, 7, 8, 9, 10, 11, 12, 13, 14, 15	2, 3, 4, 5, 6, 7, 8, 9, 10, 11, 12, 13, 14, 15
	ACAM-J8	2, 3, 11	2, 3, 11	2, 3, 4, 5, 6, 7, 8, 9, 10, 11, 12, 13, 14	2, 3, 4, 5, 6, 7, 8, 9, 10, 11, 12, 13, 14

bioRxiv preprint doi: https://doi.org/10.1101/2025.11.12.688050; this version posted November 13, 2025. The copyright holder for this preprint (which was not certified by peer review) is the author/funder, who has granted bioRxiv a license to display the preprint in perpetuity. It is made available under aCC-BY 4.0 International license.

References

1. T. Sparby, M. D. Sacchet, Toward a unified account of advanced concentrative absorption meditation: A systematic definition and classification of jhāna. *Mindfulness* **15**, 1375–1394 (2024).
2. B. Anālayo, A brief history of Buddhist absorption. *Mindfulness* **11**, 571-586 (2019).
3. L. Brasington, *Right concentration: A practical guide to the Jhanas*. (Shambala Publications, Boston, MA, 2015).
4. S. Snyder, T. Rasmussen, *Practicing the Jhanas: Traditional concentration meditation as presented by the Venerable Pa Auk Sayadaw*. (Shambala Publications, Boston, MA, 2009).
5. N. E. F. Quli, Multiple buddhist modernisms: Jhāna in convert Theravāda. *Pacific World* **10**, 225-249 (2008).
6. R. E. Buswell *et al.*, *The Princeton Dictionary of Buddhism*. (Princeton University Press, Princeton, NJ, 2014).
7. R. Shankman, *The Experience of Samadhi: An In-depth Exploration of Buddhist Meditation*. (Shambhala Publications, Inc., Boston, MA., 2008).
8. A. Brahm, *Mindfulness, Bliss, and Beyond: A Meditator's Handbook*. (Wisdom Publications, Inc, MA, MA, 2005).
9. T.-f. Kuan, Cognitive operations in Buddhist meditation: interface with Western psychology. *Contemporary Buddhism* **13**, 35-60 (2012).
